# Regioselective green synthesis of some novel indole-substituted 1*H*-benzo[*f*]chromenes *via* one-pot three-component reactions in water–ethanol media

**DOI:** 10.1039/d6ra02182d

**Published:** 2026-04-02

**Authors:** Amirhossein Khanmohammadi, Abolfazl Olyaei, Mahdieh Sadeghpour

**Affiliations:** a Department of Chemistry, Faculty of Science, Imam Khomeini International University Qazvin Iran Olyaei_a@sci.ikiu.ac.ir; b Department of Chemistry, Qa.C, Islamic Azad University Qazvin Iran

## Abstract

A regioselective, efficient and environmentally friendly method was developed for the synthesis of novel indole-substituted 1*H*-benzo[*f*]chromene derivatives through one-pot multicomponent condensation reaction of 3-cyanoacetyl indole, 2,7-dihydroxynaphthalene, and various aryl, heteroaryl and aliphatic aldehydes in EtOH/H_2_O under reflux conditions. The domino reaction proceeded *via* a piperidine-catalyzed Knoevenagel condensation between 3-cyanoacetyl indole and an aldehyde, followed by a Michael addition of 2,7-dihydroxynaphthalene and subsequent intramolecular heteroannulation. The attractive features of this method are operational simplicity, regioselectivity, green process, broad substrate scope, metal-free, shorter reaction time, easy workup, good to excellent yields and easy purification of products without utilization of any chromatography. The structures of the compounds were confirmed by FT-IR, ^1^H-NMR, and ^13^C-NMR spectroscopy and mass spectrometry.

## Abstract 2

An efficient procedure for the regioselective green synthesis of novel indole-substituted chromenes was developed. The domino reaction involves piperidine-catalyzed Knoevenagel condensation, Michael addition, and intramolecular heteroannulation.

## Graphical abstract

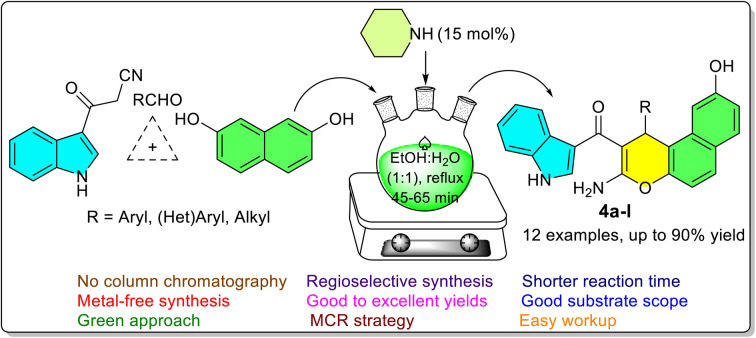

## Introduction

Chromene, benzochromene and their derivatives have been considered as an important class of oxygen-containing heterocycles. There has been increasing interest in the study of chromenes and benzochromenes due to their biological and pharmacological activities which include anti-cancer,^1–4^ anti-microbial,^5–7^ anti-influenza virus,^8^ anti-inflammatory effects,^9^ antiproliferative,^10^ antitubercular,^11^ antioxidant,^12^ anti-leishmanial,^13^ vascular disrupting^14^ and oestrogenic, anticoagulant and antispasmolytic^15^ activities. Among several synthesized chromene compounds, benzo[*f*]chromenes represent an important category of classical molecules and photochromic compounds.^16^ There has been considerable interest in chromenes and their benzo-derivatives because of their value for a variety of industrial, biological and chemical uses.^17–19^ Furthermore, 1*H*-benzo[*f*]chromene scaffolds have emerged as promising lead candidates for anticancer drug development due to their ability to target critical signaling pathways involved in cancer cell proliferation. These compounds have demonstrated diverse and potent anticancer mechanisms, including acting as *c*-Src kinase inhibitors and proapoptotic agents.^20^ They have also shown broad cytotoxic and apoptotic effects across a range of human cancer cell lines.^21^ Furthermore, others have been found to induce cell cycle arrest and apoptosis through the dual inhibition of topoisomerases and tubulin.^22,23^ Additionally, they have been utilized as powerful molecules that have apoptotic impacts with DNA binding attributes *via* an assortment of cell types.^24^

**Scheme 1:**
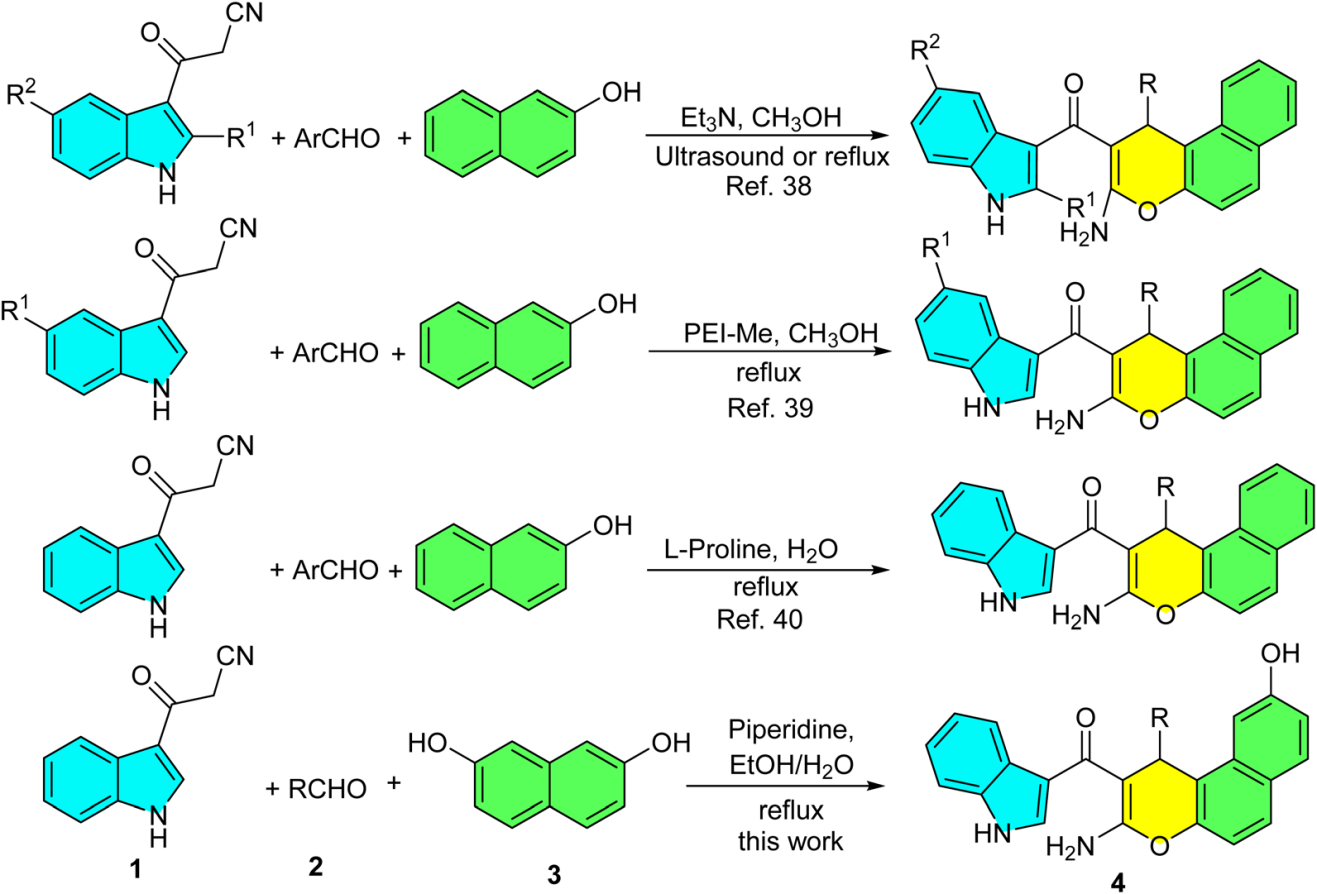
Synthesis of indole-substituted chromene derivatives.

Various synthetic routes have been developed for the construction of benzo[*f*]chromene derivatives. A prominent and widely used approach employs 2-naphthols as starting materials, which undergo a reaction with aldehydes and active methylene compounds under diverse catalytic or solvent conditions.^25^ Recent synthetic approaches for benzo[*f*]chromene derivatives has been reported. These include multi-component reactions such as: the condensation of 2-naphthol with α,β-unsaturated aldehydes and chiral 1-phenylethylamine;^26^ the reaction of 2-hydroxy-1-naphthaldehyde with indole derivatives and malononitrile catalyzed by Baker's yeast; ^27^ and the coupling of 2-naphthol with triphenylphosphine and an acetylenic ester in the presence of β-cyclodextrin.^28^ Additionally, methods using AuCl_3_/3AgOTf to catalyze the reaction of 2-naphthol with ketones have been developed.^29^ Additional synthetic strategies include the reaction of 2-naphthol with acetophenone derivatives and triethyl orthobenzoate using a bis[7-*tert*-butyl-2-anilinotropone]Ti complex;^30^ the ethylene diamine diacetate-catalyzed condensation of 2-naphthol with 3-methylbut-2-enal;^31^ and the CuCl_2_-mediated reaction of β-oxodithioesters and an *S*,*S*-diacetal with 2-hydroxy-1-naphthaldehyde.^32^ Other notable methods involve microwave-assisted reactions of 7-methoxy-2-naphthol with aromatic aldehydes and 2-cyanoethanethioamide;^33^ the reaction of 2-naphthol with aromatic aldehydes and 3,3,3-trifluoro-1-phenylpropan-1-one in 1,4-disulfo-1,4-diazoniabicyclo[2.2.2]octane chloride (DSDABCO),^34^ the use of In(OTf)_3_ to catalyze the coupling of β-naphthols with enals;^35^ the Kit-6-NH_2_@Schiff base complex promoted reaction of 2-naphthol with aldehyde and 4,4,4-trifluoro1-phenyl-1,3-butanedione,^36^ the NiFe_2_O_4_@Silicapropyl magnetic nanoparticles-catalyzed condensation of aromatic aldeydes with naphthalen-2-ol, and 4,4,4-trifluoro-1-phenylbutane1,3-dione.^37^ Moreover, indole-substituted chromene derivatives were obtained from the reaction of 2-naphthol with 3-cyanoacetylindoles and aryl aldehydes. This transformation employed Et_3_N^38^ and PEI-Me^39^ as catalysts in methanol, as well as l-proline in aqueous medium (Scheme 1).^40^ These compounds exhibit antibacterial, anti-inflammatory, and analgesic activities. Notably, they act as highly specific NorA efflux pump inhibitors, helping to mitigate drug-resistant strains of *S. aureus*. As shown in Scheme 1, these reactions were carried out either in methanol (a toxic solvent) with an expensive catalyst, or in water under reflux conditions with prolonged reaction times. Based on the established biological significance of chromene scaffolds and in continuation of our previous study on 3-cyanoacetyl indoles^41^ and multi-component synthetic methodologies,^42^ in the present work, we report a facial one-pot, three-component domino reaction of 3-cyanoacetyl indole, 2,7-dihydroxynaphthalene, various aryl, heteroaryl and aliphatic aldehydes using piperidine as an inexpensive catalyst in EtOH/H_2_O under heating conditions to afford the corresponding novel indole-substituted 1*H*-benzo[*f*]chromene derivatives in good to excellent yields. Furthermore, based on the biological properties of these compounds, the newly synthesized derivatives may also exhibit promising novel biological activities.

## Results and discussion

**Scheme 2:**
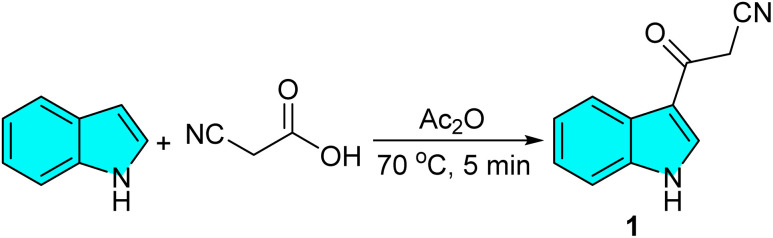
Synthesis of 3-cyanoacetyl indole (1).

To initiate our study, 3-cyanoacetyl indole (1) was achieved *via* the reaction of indole and cyanoacetic acid in Ac_2_O at 70 °C for 5 min (Scheme 1). The identity of compound 1 was confirmed by comparison of its physical and spectral data with those reported in reference (Scheme 2).^43^ Then, the reaction of 3-cyanoacetyl indole (1) (1.0 mmol), 4-nitrobenzaldehyde 2a (1.0 mmol), and 2,7-dihydroxynaphthalene 3 (1.0 mmol) was chosen as a model (Table 1). First, carrying out this model reaction in the absence of a catalyst in ethanol at room temperature for 24 h yielded no product (Table 1, entry 1).

**Table 1:** Optimization of the reaction conditions^a^

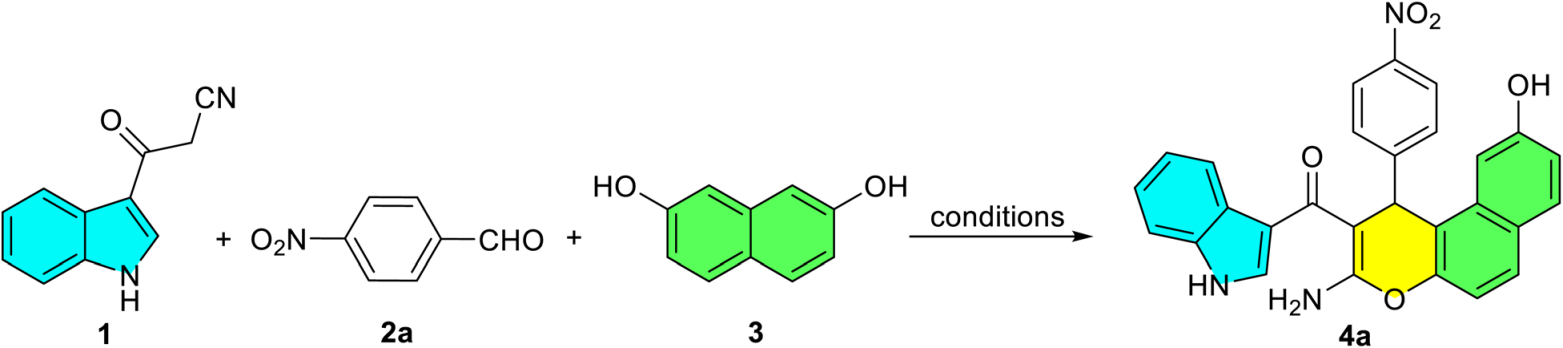					
Entry	Solvent	Temperature (^o^C)	Catalyst loading (mol%)	Time (h)	Yield^b^ (%)
1	EtOH	r.t	—	24	—
2	EtOH	Reflux	—	8	32
3	EtOH	Reflux	PTSA (10%)	8	Trace
4	EtOH	Reflux	Et3N (10%)	4	52
5	EtOH	Reflux	Piperidine (10%)	3	58
6	MeOH	Reflux	Piperidine (10%)	3	56
7	HOAc	Reflux	—	8	Trace
8	CH3CN	Reflux	Piperidine (10%)	6	51
9	CH2Cl2	Reflux	Piperidine (10%)	8	43
10	CHCl3	Reflux	Piperidine (10%)	8	47
11	Toluene	Reflux	Piperidine (10%)	1.5	50
12	H2O	Reflux	Piperidine (10%)	8	31
13	EtOH + H2O (1 : 1)	Reflux	Piperidine (10%)	1.5	65
14	EtOH + H2O (2 : 1)	Reflux	Piperidine (10%)	1.5	52
15	EtOH + H2O (1 : 2)	Reflux	Piperidine (10%)	1.5	45
16	EtOH + H2O (1 : 1)	Reflux	Piperidine (5%)	2	55
17	EtOH + H2O (1 : 1)	Reflux	Piperidine (15%)	1	79
18	EtOH + H2O (1 : 1)	Reflux	Piperidine (20%)	1	80
19	—	80	Piperidine (15%)	1	52
20	—	100	Piperidine (15%)	1	54

a All the reactions were performed with 1 (1 mmol), 2a (1 mmol), 3 (1 mmol), and solvent (10 mL).

b Isolated yields.

When the reaction was stirred for 8 h in ethanol under reflux conditions without using any catalyst, the expected (3-amino-9-hydroxy-1-(4-nitrophenyl)-1*H*-benzo[*f*]chromen-2-yl)(1*H*-indol-3-yl)methanone (4a) was obtained in only 32% yield (Table 1, entry 2). We then investigated the reaction using *p-*TSA (10 mol%) in ethanol under reflux. Only trace amount of product 4a was formed (Table 1, entry 3). Next, we investigated base catalysts to optimize the yield and reaction time. The model reaction with Et_3_N (10 mol%) afforded product 4a in 52% yield, while piperidine (10 mol%) gave an improved yield of 58% in refluxing EtOH (Table 1, entries 4–5). Piperidine as a base catalyst seemed to be more efficient for the above transformation. Using piperidine (10 mol%) as the catalyst under reflux, the model reaction was evaluated in a series of protic and aprotic solvents (Table 1, entries 6–15). The protic solvents tested were MeOH, H_2_O HOAc, and EtOH/H_2_O mixtures (1 : 1, 2 : 1, 1 : 2). The aprotic solvents were CH_3_CN, CH_2_Cl_2_, CHCl_3_, and toluene. The results revealed that using EtOH/H_2_O (1 : 1) provided the desired product in 65% yield after 1.5 h (Table 1, entry 13). To determine the optimal catalyst loading, reactions were performed in refluxing EtOH/H_2_O (1 : 1) using 5, 15, and 20 mol% piperidine. A loading of 5 mol% afforded the product in 55% yield after 2 h. Surprisingly, increasing the catalyst to 15 mol% significantly improved the yield to 79% within 60 minutes (Table 1, entries 16–17). No significant difference in yield was observed when the catalyst loading was increased from 15 to 20 mol% (Table 1, entry 18). The reaction was also tested under solvent-free conditions at 80 °C and 100 °C, affording 54 and 52% yields after 1 h, respectively (Table 1, entries 19–20). On the basis of all of these experiments, the optimum reaction conditions were identified as 1 : 1 (v/v) ethanol/water under reflux conditions catalyzed by piperidine (15 mol%).

With the optimal conditions established, we evaluated the substrate scope using 12 diverse aldehydes 2. These included substituted aromatic aldehydes bearing electron-withdrawing groups (EWGs) such as 4-nitrobenzaldehyde, 3-nitrobenzaldehyde, 4-chlorobenzaldehyde, 3-chlorobenzaldehyde, 4-flourobenzaldehyde, 3-flourobenzaldehyde, 3-bromobenzaldehyde and electron-donating groups (EDGs) such as 4-methylbenzaldehyde, 4-methoxybenzaldehyde, a heteroaromatic aldehyde (furfural), and an aliphatic aldehyde (butyraldehyde). The results are summarized in Table 2. As shown in Table 2, under the optimized conditions, the reaction proceeded successfully with a range of aldehydes. Substrates bearing electron-withdrawing groups (EWGs) furnished products 4a–g in good yields (75–81%) over 55–65 min. In contrast, aldehydes with electron-donating groups (EDGs) reacted more rapidly, providing products 4h–i in excellent yields (88–90%) within a shorter time (50 min). Similarly, furfural, benzaldehyde, and butyraldehyde afforded products 4j–l in high yields (75–82%) with reaction times of 45–60 min. These results indicate that the reaction rate and yield are favorably influenced by EDGs. Notably, all products could be purified by simple trituration in boiling CHCl_3_, eliminating the need for chromatography.

**Table 2:** Synthesis of indole-substituted 1*H*-benzo[*f*]chromenes catalyzed by piperidine^a^

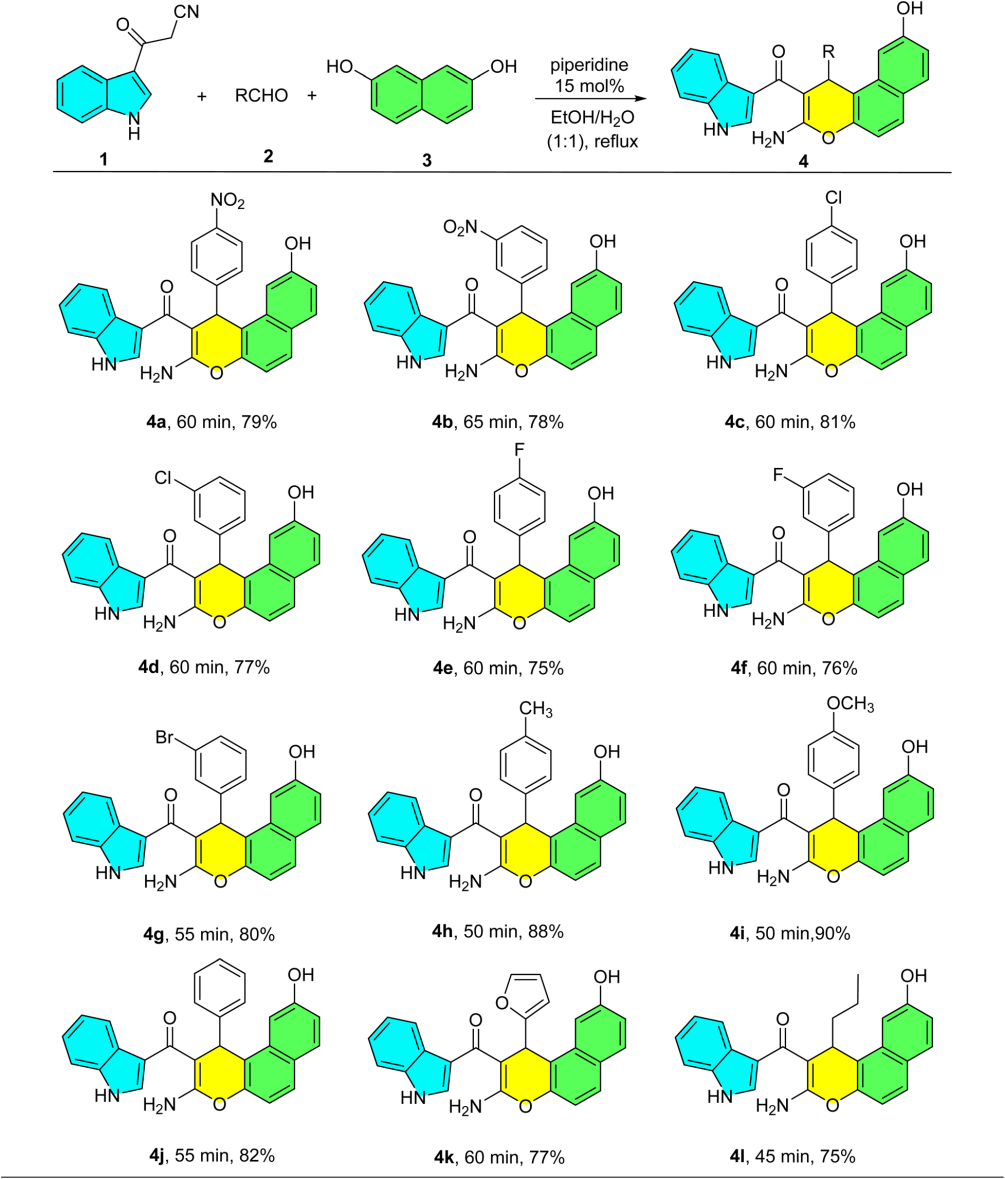

a Reaction conditions: 3-cyanoacetyl indole (1 mmol), aldehyde (1 mmol), 2,7-dihydroxynaphthalene (1 mmol) and piperidine (15 mol%) in EtOH : H_2_O (1 : 1) under reflux conditions.

To the best of our knowledge, there is no report for the synthesis of indole-substituted 1*H*-benzo[*f*]chromene derivatives through the MCRs of 3-cyanoacetyl indole, 2,7-dihydroxynaphthalene, various aryl, heteroaryl and aliphatic aldehydes. Therefore, all the synthesized compounds were unknown, and were characterized by Fourier Transform Infrared (FT-IR), ^1^H and ^13^C-NMR, mass spectrometry analysis and melting points. The presence of the expected functional groups was confirmed by infrared spectroscopy. In the IR spectrum of compound 4a, stretching vibrations were observed for N–H and O–H groups (3501, 3451, 3128 cm^−1^), a carbonyl group (1637 cm^−1^), and a nitro group (1522 and 1340 cm^−1^). In the ^1^H NMR spectrum of 4a, the methine proton appeared as a singlet at *δ* 6.00 ppm. Aromatic protons resonated as a multiplet in the range *δ* 6.85–7.92 ppm. Three exchangeable proton singlets were observed at *δ* 8.70 (NH_2_), 9.79 (OH), and 11.66 ppm (NH). Upon addition of D_2_O to the NMR sample, the signals corresponding to the exchangeable protons were eliminated due to deuterium exchange. The ^13^C-NMR spectrum of 4a displayed signals corresponding to the methine carbon (*δ* 38.3 ppm), the carbonyl group (*δ* 188.0 ppm), and aromatic carbons (*δ* 88.8–162.0 ppm).

Mass spectral data for compounds 4a–k supported their structures, showing low-intensity molecular ions consistent with facile fragmentation. For compound 4a, the mass spectrum showed a molecular ion peak at *m*/*z* 477. This fragmented to yield key ions at *m*/*z* 355, 184, 144, 131, 116, and 103, with the base peak appearing at *m*/*z* 160.

**Scheme 3:**
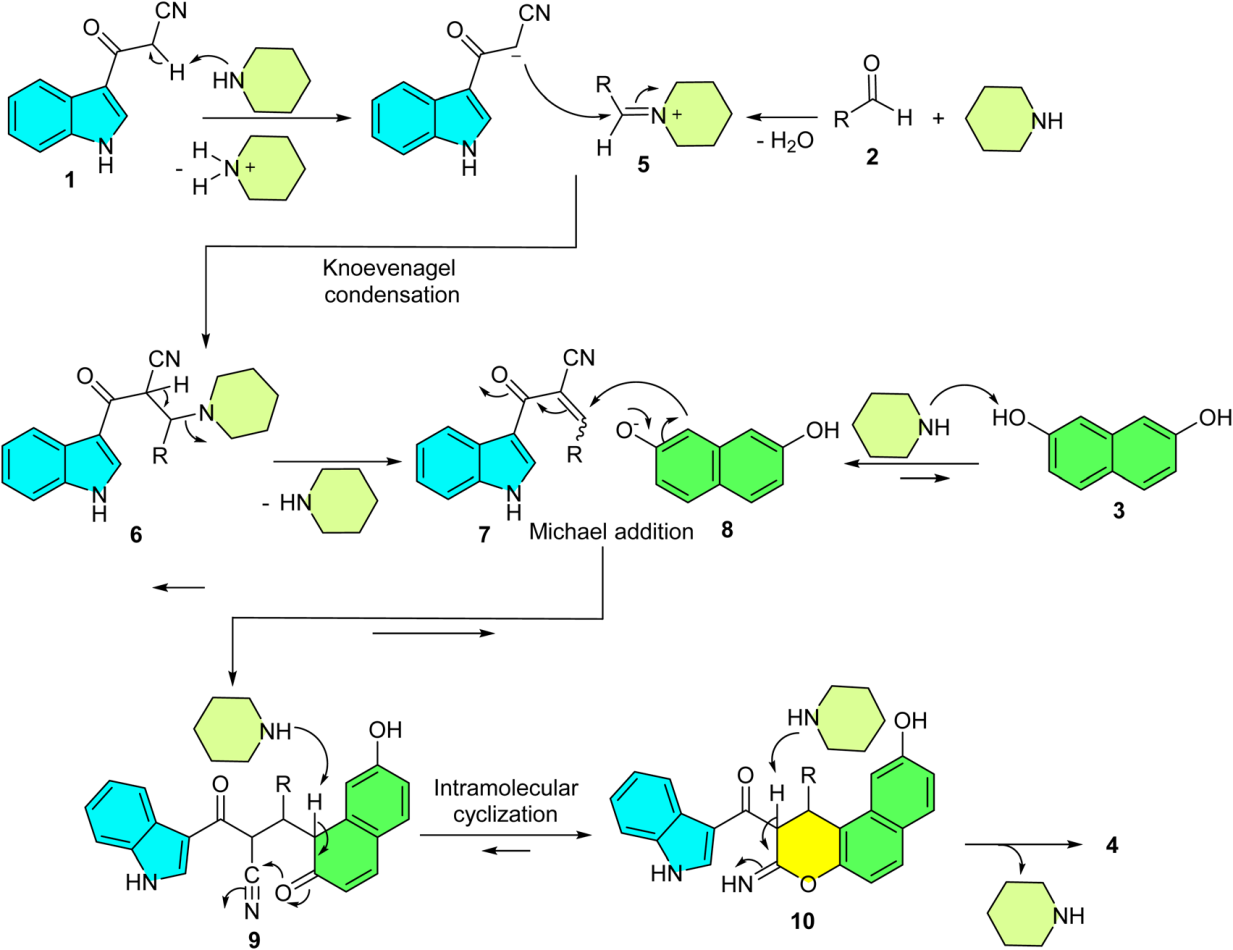
Proposed mechanism for the synthesis of indole-substituted 1*H*-benzo[*f*]chromene derivatives 4.

Based on comprehensive experimental evidence, we propose a plausible reaction mechanism for the formation of glycine derivatives 4, as illustrated in Scheme 3. In the first step, a piperidine-mediated Knoevenagel condensation of 3-cyanoacetyl indole (1) and iminium ion intermediate 5 formed by condensation of piperidine with the aldehyde 2 gives Knoevenagel adduct 7. Deprotonation of 2,7-dihydroxynaphthalene (3) by piperidine forms the nucleophile 8, which adds to 7 in a Michael reaction to yield adduct 9. This is followed by an intramolecular *O*-cyclization, where the enolate moiety of 9 attacks the electrophilic nitrile carbon, forming intermediate 10. Tautomerization of 10 then provides the final product 4. The proposed mechanism was confirmed by studying the order of addition of reagents. To support the proposed mechanism, we adopted a two-component experimental approach. When 2,7-dihydroxynaphthalene (3) was reacted with aldehyde 2 under optimized conditions, no product was detected even after 2 hours. However, conducting the reaction between 1 and 2 under similar conditions led to the formation of intermediate 7 within 30 minutes. Subsequent addition of 3 to this mixture yielded the final product. These results clearly indicate that the reaction proceeds *via in situ* formation of the adduct 7.

## Conclusions

In summary, we have described piperidine-catalyzed heteroannulation as an alternative method for the synthesis of a series of indole-substituted 1*H*-benzo[*f*]chromene derivatives 4 in a regiochemical manner by the reaction of 3-cyanoacetyl indole, aldehyde, and 2,7-dihydroxynaphthalene under conventional condition. This reaction involving Knoevenagel condensation, Michael addition and ring closure. The proposed protocol offers several advantages, including good to excellent yields, a broad substrate scope, the one-step conversion of simple, readily available starting materials into an interesting class of fused heterocyclic scaffolds of benzochromene derivatives. The procedure also features straightforward workup and product purification, which avoids the need for chromatographic methods. Studies to extend the reaction scope and explore further synthetic applications of this methodology are currently underway in our laboratory.

## Experimental methods and materials

### General information

All commercially available chemicals and reagents were used without further purification. Melting points were determined with Gallenkamp MFB.595.010M apparatus. FT-IR spectra were recorded on a Bruker Tensor 27 spectrophotometer. The ^1^H-and ^13^C-NMR spectra were recorded in DMSO-*d*_6_ on Bruker DRX-300 Avance spectrometers. Chemical shifts (*δ*) are reported in parts per million and are referenced to the NMR solvent. Mass spectra of the products were obtained with a HP (Agilent technologies) 5973 Mass Selective Detector.

#### General procedure for the synthesis of 4a–l

A mixture of 3-cyanoacetyl indole (1, 1 mmol), aldehyde (2, 1 mmol), 2,7-dihydroxynaphthalene (3, 1 mmol) and piperidine (15 mol%) in EtOH : H_2_O (1 : 1) (10 ml) was stirred at reflux for an appropriate time (Table 2), and the progress of the reaction was monitored by TLC (*n*-hexane/ethyl acetate: 1 : 1). Upon completion of the reaction, the mixture was cooled to room temperature. The solid product was collected by filtration, washed with cold H_2_O/EtOH, and dried. For further purification, each precipitate was suspended in boiling CHCl_3_ and stirred for 5 minutes. After cooling, the solids were collected by filtration, washed with cold CHCl_3_, and dried to obtain pure products 4a–l in good to excellent yields. All compounds were fully characterized by FT-IR, ^1^H and ^13^C-NMR spectroscopy, and mass spectrometry.

#### (3-Amino-9-hydroxy-1-(4-nitrophenyl)-1*H*-benzo[*f*]chromen-2-yl)(1*H*-indol-3-yl)methanone (4a)

Yellow powder; M.P. = 255–256 °C; IR (KBr) (*υ*_max_ cm^−1^): 3501, 3451, 3128, 3040, 2916, 1637, 1604, 1522, 1479, 1447, 1429, 1340, 1206, 1136, 1086; ^1^H-NMR (300 MHz, DMSO-*d*_6_): *δ*_H_ 6.00 (s, 1H, methine-H), 6.85–6.98 (m, 5H, Ar–H), 7.13–7.19 (m, 2H, Ar–H), 7.48–7.52 (m, 2H, Ar–H), 7.73–7.92 (m, 5H, Ar–H), 8.70 (s, 2H, NH_2_), 9.79 (s, 1H, OH), 11.66 (s, 1H, NH); ^1^H-NMR (300 MHz, DMSO-*d*_6_ + D_2_O): *δ*_H_ 5.93 (s, 1H, methine-H), 6.71–6.94 (m, 5H, Ar–H), 7.13–7.22 (m, 2H, Ar–H), 7.34–7.38 (m, 1H, Ar–H), 7.52 (d, 1H, *J* = 8.1 Hz, Ar–H), 7.63–7.85 (m, 5H, Ar–H); ^13^C-NMR (75 MHz, DMSO-*d*_6_): *δ*_C_ 38.30 (methine-C), 88.85, 105.03, 112.61, 113.61, 117.28, 117.69, 117.93, 120.41, 120.89, 122.48, 124.19, 125.69, 128.05, 128.52, 129.63, 130.88, 131.55, 132.45, 136.41, 146.13, 147.97, 154.58, 157.07, 162.00, 188.02 (C=O); MS (EI, 70 ev) *m*/*z* (%): 477 [M]^+^, 355, 184, 160 (100), 144, 131, 116, 103, 89, 77, 63, 51.

#### (3-Amino-9-hydroxy-1-(3-nitrophenyl)-1*H*-benzo[*f*]chromen-2-yl)(1*H*-indol-3-yl)methanone (4b)

Yellow powder; M.P. = 182–183 °C; IR (KBr) (*υ*_max_ cm^−1^): 3416, 3253, 3128, 3064, 2927, 1636, 1603, 1528, 1483, 1436, 1385, 1349, 1230, 1200, 1136, 1080; ^1^H-NMR (300 MHz, DMSO-*d*_6_): *δ*_H_ 6.00 (s, 1H, methine-H), 6.84 (d, 1H, *J* = 2.3, Ar–H), 6.92–6.97 (m, 2H, Ar–H), 7.06 (d, 1H, *J* = 7.7, Ar–H), 7.13–7.21 (m, 2H, Ar–H), 7.30–7.52 (m, 4H, Ar–H), 7.72–7.85 (m, 4H, Ar–H), 8.72 (s, 2H, NH_2_), 9.79 (s, 1H, OH), 11.67 (s, 1H, NH); ^13^C-NMR (75 MHz, DMSO-*d*_6_): *δ*_C_ 38.06 (methine-C), 89.22, 104.98, 112.61, 113.59, 117.20, 117.72, 118.04, 120.26, 120.86, 121.33, 121.62, 122.47, 123.74, 125.55, 125.73, 128.29, 129.69, 130.39, 130.92, 132.40, 133.48, 136.43, 148.03, 149.23, 157.10, 162.00, 188.20 (C=O); MS (EI, 70 ev) *m*/*z* (%): 477 [M]^+^, 184, 160 (100), 144, 131, 117, 116, 102, 103, 89, 77, 63, 51.

#### (3-Amino-1-(4-chlorophenyl)-9-hydroxy-1*H*-benzo[*f*]chromen-2-yl)(1*H*-indol-3-yl)methanone (4c)

White powder; M.P. = 217–218 °C; IR (KBr) (*υ*_max_ cm^−1^): 3426, 3247, 3046, 2981, 1635, 1600, 1552, 1488, 1441, 1380, 1306, 1239, 1219, 1199, 1097; ^1^H-NMR (300 MHz, DMSO-*d*_6_): *δ*_H_ 5.88 (s, 1H, methine-H), 6.68 (d, 2H, *J* = 8.4, Ar–H), 6.89–6.97 (m, 3H, Ar–H), 7.07–7.17 (m, 4H, Ar–H), 7.47–7.54 (m, 2H, Ar–H), 7.69 (d, 1H, *J* = 2.7 Hz, Ar–H), 7.74–7.79 (m, 2H, Ar–H), 8.67 (s, 2H, NH_2_), 9.76 (s, 1H, OH), 11.62 (s, 1H, NH); ^13^C-NMR (75 MHz, DMSO-*d*_6_): *δ*_C_ 37.54 (methine-C), 89.55, 105.19, 112.50, 113.57, 117.60, 117.97, 118.28, 120.58, 120.82, 122.42, 125.72, 125.79, 128.36, 128.67, 128.77, 129.19, 130.77, 130.98, 132.45, 136.36, 146.06, 147.93, 156.92, 162.16, 188.04 (C=O); MS (EI, 70 ev) *m*/*z* (%): 468 [M + 2]^+^, 466 [M]^+^, 355, 308, 306, 279, 242, 184, 160, 144 (100), 131, 116, 89, 76, 63.

#### (3-Amino-1-(3-chlorophenyl)-9-hydroxy-1*H*-benzo[*f*]chromen-2-yl)(1*H*-indol-3-yl)methanone (4d)

White powder; M.P. = 210–211 °C; IR (KBr) (*υ*_max_ cm^−1^): 3424, 3246, 3065, 2926, 1635, 1602, 1520, 1478, 1441, 1392, 1302, 1239, 1219, 1198, 1080; ^1^H-NMR (300 MHz, DMSO-*d*_6_): *δ*_H_ 5.89 (s, 1H, methine-H), 6.59–6.64 (m, 2H, Ar–H), 6.87–6.98 (m, 3H, Ar–H), 7.05–7.17 (m, 4H, Ar–H), 7.48–7.52 (m, 2H, Ar–H), 7.70 (d, 1H, *J* = 2.6 Hz, Ar–H), 7.78 (t, 2H, *J* = 8.7 Hz, Ar–H), 8.67 (s, 2H, NH_2_), 9.78 (s, 1H, OH), 11.65 (s, 1H, NH); ^13^C-NMR (75 MHz, DMSO-*d*_6_): *δ*_C_ 37.90 (methine-C), 89.42, 105.12, 112.51, 113.57, 117.66, 117.88, 117.99, 120.48, 120.85, 122.45, 125.50, 125.73, 126.49, 126.60, 128.30, 129.35, 130.77, 130.83, 132.45, 133.30, 136.40, 148.02, 149.53, 153.20, 156.98, 162.13, 188.08 (C=O); MS (EI, 70 ev) *m*/*z* (%): 468 [M + 2]^+^, 466 [M]^+^, 308, 306, 279, 184, 160 (100), 144, 131, 116, 103, 89, 77, 63.

#### (3-Amino-1-(4-fluorophenyl)-9-hydroxy-1*H*-benzo[*f*]chromen-2-yl)(1*H*-indol-3-yl)methanone (4e)

Cream powder; M.P. = 217–218 °C; IR (KBr) (*υ*_max_ cm^−1^): 3431, 3244, 3047, 2925, 1637, 1596, 1523, 1508, 1482, 1437, 1407, 1377, 1309, 1238, 1199, 1136, 1080; ^1^H-NMR (300 MHz, DMSO-*d*_6_): *δ*_H_ 5.89 (s, 1H, methine-H), 6.68–6.73 (m, 2H, Ar–H), 6.84–6.97 (m, 5H, Ar–H), 7.12–7.17 (m, 2H, Ar–H), 7.46–7.55 (m, 2H, Ar–H), 7.68 (d, 1H, *J* = 2.7 Hz, Ar–H), 7.74–7.79 (m, 2H, Ar–H), 8.66 (s, 2H, NH_2_), 9.75 (s, 1H, OH), 11.62 (s, 1H, NH); ^13^C-NMR (75 MHz, DMSO-*d*_6_): *δ*_C_ 37.31 (methine-C), 89.91, 105.26, 112.49, 113.60, 115.34, 115.68 117.58, 117.98, 118.68, 120.62, 120.81, 122.42, 125.73, 125.84, 128.31, 128.53, 128.66, 129.10, 130.78, 132.46, 136.36, 143.29, 147.94, 156.89, 158.91, 162.26, 188.07 (C=O); MS (EI, 70 ev) *m*/*z* (%): 450 [M]^+^, 307, 290, 263, 224, 184, 160, 144 (100), 131, 116, 89, 63.

#### (3-Amino-1-(3-fluorophenyl)-9-hydroxy-1*H*-benzo[*f*]chromen-2-yl)(1*H*-indol-3-yl)methanone (4f)

White powder; M.P. = 206–208 °C; IR (KBr) (*υ*_max_ cm^−1^): 3425, 3247, 3051, 2925, 1636, 1602, 1522, 1483, 1441, 1390, 1301, 1243, 1200, 1137, 1081; ^1^H-NMR (300 MHz, DMSO-*d*_6_): *δ*_H_ 5.90 (s, 1H, methine-H), 6.33–6.37 (m, 1H, Ar–H), 6.55 (d, 1H, *J* = 7.7 Hz, Ar–H), 6.78–6.98 (m, 4H, Ar–H), 7.04–7.17 (m, 3H, Ar–H), 7.47–7.53 (m, 2H, Ar–H), 7.69 (d, 1H, *J* = 2.7 Hz, Ar–H), 7.78 (t, 2H, *J* = 7.9 Hz, Ar–H), 8.68 (s, 2H, NH_2_), 9.76 (s, 1H, OH), 11.64 (s, 1H, NH); ^13^C-NMR (75 MHz, DMSO-*d*_6_): *δ*_C_ 37.85 (methine-C), 89.46, 105.19, 112.50, 113.10, 113.29, 113.44, 113.58, 117.64, 117.97, 118.09, 120.54, 120.84, 122.44, 122.90, 125.72, 125.79, 128.29, 129.27, 130.79, 130.88, 132.48, 136.38, 148.01, 149.91, 150.01, 156.95, 160.41, 162.24, 164.29, 188.06 (C=O); MS (EI, 70 ev) *m*/*z* (%): 450 [M]^+^, 307, 290, 263, 224, 184, 160, 144 (100), 131, 116, 103, 89, 76, 63.

#### (3-Amino-1-(3-bromophenyl)-9-hydroxy-1*H*-benzo[*f*]chromen-2-yl)(1*H*-indol-3-yl)methanone (4g)

White powder; M.P. = 203–204 °C; IR (KBr) (*υ*_max_ cm^−1^): 3423, 3255, 3057, 2925, 1636, 1602, 1520, 1480, 1440, 1385, 1238, 1216, 1198, 1136, 1080; ^1^H-NMR (300 MHz, DMSO-*d*_6_): *δ*_H_ 5.88 (s, 1H, methine-H), 6.65 (d, 1H, *J* = 7.7 Hz, Ar–H), 6.74–7.02 (m, 5H, Ar–H), 7.13–7.19 (m, 3H, Ar–H), 7.48–7.52 (m, 2H, Ar–H), 7.70 (d, 1H, *J* = 2.4 Hz, Ar–H), 7.78 (t, 2H, *J* = 8.7 Hz, Ar–H), 8.67 (s, 2H, NH_2_), 9.78 (s, 1H, OH), 11.65 (s, 1H, NH); ^13^C-NMR (75 MHz, DMSO-*d*_6_): *δ*_C_ 37.91 (methine-C), 89.46, 105.11, 112.53, 113.57, 117.68, 117.85, 118.00, 119.07, 120.48, 120.87, 122.08, 122.46, 125.72, 125.85, 128.30, 129.38, 129.50, 130.84, 131.07, 132.44, 136.39, 148. 32, 149.79, 156.99, 160.13, 162.11, 188.08 (C=O); MS (EI, 70 ev) *m*/*z* (%): 512 [M + 2]^+^, 510 [M]^+^, 352, 350, 325, 323, 271, 242, 184, 160 (100), 144, 131, 116, 103, 89, 77, 63.

#### (3-Amino-9-hydroxy-1-(*p*-tolyl)-1*H*-benzo[*f*]chromen-2-yl)(1*H*-indol-3-yl)methanone (4h)

White powder; M.P. = 225–226 °C; IR (KBr) (*υ*_max_ cm^−1^): 3427, 3249, 3047, 2924, 1635, 1601, 1523, 1482, 1440, 1385, 1240, 1219, 1199, 1137; ^1^H-NMR (300 MHz, DMSO-*d*_*6*_): *δ*_H_ 2.07 (s, 3H, CH_3_), 5.85 (s, 1H, methine-H), 6.62 (d, 2H, *J* = 7.9 Hz, Ar–H), 6.83 (d, 2H, *J* = 7.9 Hz, Ar–H), 6.92–6.98 (m, 3H, Ar–H), 7.11–7.17 (m, 2H, Ar–H), 7.47 (d, 1H, *J* = 8.1 Hz, Ar–H), 7.57 (d, 1H, *J* = 7.9 Hz, Ar–H), 7.66 (d, 1H, *J* = 2.6 Hz, Ar–H), 7.73–7.76 (dd, 2H, *J* = 8.6, 2.2 Hz, Ar–H), 8.64 (s, 2H, NH_2_), 9.73 (s, 1H, OH), 11.59 (s, 1H, NH); ^13^C-NMR (75 MHz, DMSO-*d*_6_): *δ*_C_ 20.85 (CH_3_), 37.60 (methine-C), 90.10, 101.61, 105.43, 112.44, 113.57, 117.50, 118.04, 119.15, 120.76, 122.38, 125.73, 126.00, 126.78, 128.27, 128.83, 129.35, 130.69, 132.55, 135.46, 136.35, 144.24, 147.91, 156.76, 162.34, 188.08 (C=O); MS (EI, 70 ev) *m*/*z* (%): 446 [M]^+^, 286, 259, 242, 184, 160 (100), 144, 131, 116, 103, 89, 76, 63.

#### (3-Amino-9-hydroxy-1-(4-methoxyphenyl)-1*H*-benzo[*f*]chromen-2-yl)(1*H*-indol-3-yl)methanone (4i)

White powder; M.P. = 205–207 °C; IR (KBr) (*υ*_max_ cm^−1^): 3424, 3249, 3060, 2930, 2838, 1636, 1599, 1578, 1522, 1511, 1482, 1437, 1387, 1252, 1240, 1200, 1136; ^1^H-NMR (300 MHz, DMSO-*d*_6_): *δ*_H_ 3.55 (s, 3H, OCH_3_), 5.84 (s, 1H, methine-H), 6.59 (d, 2H, *J* = 8.9 Hz, Ar–H), 6.66 (d, 2H, *J* = 8.9 Hz, Ar–H), 6.93–6.98 (m, 3H, Ar–H), 7.11–7.16 (m, 2H, Ar–H), 7.47 (d, 1H, *J* = 8.1 Hz, Ar–H), 7.58 (d, 1H, *J* = 8.0 Hz, Ar–H), 7.66 (d, 1H, *J* = 2.6 Hz, Ar–H), 7.75 (d, 2H, *J* = 8.7 Hz, Ar–H), 8.65 (s, 2H, NH_2_), 9.74 (s, 1H, OH), 11.59 (s, 1H, NH); ^13^C-NMR (75 MHz, DMSO-*d*_6_): *δ*_C_ 37.08 (methine-C), 55.33 (OCH_3_), 90.26, 105.43, 112.10, 112.43, 113.60, 114.19, 117.51, 118.02, 119.35, 120.79, 122.40, 125.75, 126.02, 127.86, 128.27, 128.79, 130.71, 132.53, 136.36, 139.25, 147.90, 156.79, 157.85, 162.42, 188.08 (C=O); MS (EI, 70 ev) *m*/*z* (%): 462 [M]^+^, 302, 259, 242, 184, 160 (100), 144, 131, 116, 103, 89, 77, 63.

#### (3-Amino-9-hydroxy-1-phenyl-1*H*-benzo[*f*]chromen-2-yl)(1*H*-indol-3-yl)methanone (4j)

White powder; M.P. = 210–211 °C; IR (KBr) (*υ*_max_ cm^−1^): 3431, 3245, 3056, 2924, 1635, 1600, 1522, 1482, 1440, 1391, 1240, 1219, 1199, 1137; ^1^H-NMR (300 MHz, DMSO-*d*_6_): *δ*_H_ 5.89 (s, 1H, methine-H), 6.73 (d, 2H, *J* = 7.2 Hz, Ar–H), 6.95–7.05 (m, 6H, Ar–H), 7.12–7.16 (m, 2H, Ar–H), 7.47 (d, 1H, *J* = 8.0 Hz, Ar–H), 7.56 (d, 1H, *J* = 8.0 Hz, Ar–H), 7.67 (d, 1H, *J* = 2.6 Hz, Ar–H), 7.74–7.78 (m, 2H, Ar–H), 8.65 (s, 2H, NH_2_), 9.73 (s, 1H, OH), 11.60 (s, 1H, NH); ^13^C-NMR (75 MHz, DMSO-*d*_6_): *δ*_C_ 37.98 (methine-C), 90.00, 105.37, 112.44, 113.58, 117.53, 118.03, 118.95, 120.73, 122.38, 125.74, 125.94, 126.43, 126.87, 128.28, 128.84, 130.72, 132.55, 136.36, 146.16, 147.14, 148.00, 149.72, 156.80, 162.34, 188.06 (C=O); MS (EI, 70 ev) *m*/*z* (%): 432 [M]^+^, 355, 306, 238, 209, 184, 160, 144 (100), 131, 117, 116, 103, 91, 89, 77, 69, 55.

#### (3-Amino-1-(furan-2-yl)-9-hydroxy-1*H*-benzo[*f*]chromen-2-yl)(1*H*-indol-3-yl)methanone (4k)

Brown powder; M.P. = 231–232 °C; IR (KBr) (*υ*_max_ cm^−1^): 3444, 3383, 3242, 3051, 2929, 1637, 1602, 1519, 1480, 1441, 1378, 1301, 1227, 1198, 1199, 1138; ^1^H-NMR (300 MHz, DMSO-*d*_6_): *δ*_H_ 5.72 (d, 1H, *J* = 3.2 Hz, Ar–H), 5.90 (s, 1H, methine-H), 6.17–6.19 (dd, 1H, *J* = 3.1, 1.8 Hz, Ar–H), 6.95–7.01 (m, 2H, Ar–H), 7.06–7.19 (m, 3H, Ar–H), 7.35 (s, 1H, Ar–H), 7.43 (d, 1H, *J* = 8.0 Hz, Ar–H), 7.58 (d, 1H, *J* = 2.8 Hz, Ar–H), 7.69 (d, 1H, *J* = 8.0 Hz, Ar–H), 7.74–7.78 (m, 2H, Ar–H), 8.90 (s, 2H, NH_2_), 9.83 (s, 1H, OH), 11.56 (s, 1H, NH); ^13^C-NMR (75 MHz, DMSO-*d*_6_): *δ*_C_ 32.32 (methine-C), 86.44, 105.38, 110.80, 112.27, 113.52, 116.70, 117.22, 117.70, 119.23, 120.73, 121.13, 122.46, 125.62, 126.54, 127.60, 129.10, 130.64, 132.56, 136.26, 142.28, 147.98, 156.92, 163.56, 187.47 (C=O); MS (EI, 70 ev) *m*/*z* (%): 422 [M]^+^, 262, 238, 218, 197, 184, 160, 144 (100), 131, 116, 103, 89, 77, 63, 51.

#### (3-Amino-9-hydroxy-1-propyl-1*H*-benzo[*f*]chromen-2-yl)(1*H*-indol-3-yl)methanone (4l)

White powder; M.P. = 202–203 °C; IR (KBr) (*υ*_max_ cm^−1^): 3444, 3226, 3051, 2955, 2931, 2859, 1634, 1599, 1520, 1477, 1444, 1381, 1236, 1216, 1201, 1170; ^1^H-NMR (300 MHz, DMSO-*d*_6_): *δ*_H_ 0.53 (t, 3H, *J* = 7.7 Hz, CH_3_), 0.96–0.99 (m, 2H, CH_2_), 1.29–1.40 (m, 2H, CH_2_), 4.75 (t, 1H, *J* = 10.7 Hz, methine-H), 6.91–7.15 (m, 5H, Ar–H), 7.43 (d, 1H, *J* = 8.1 Hz, Ar–H), 7.62 (d, 1H, *J* = 8.0 Hz, Ar–H), 7.69–7.78 (m, 3H, Ar–H), 8.62 (s, 2H, NH_2_), 9.76 (s, 1H, OH), 11.53 (s, 1H, NH); ^13^C-NMR (75 MHz, DMSO-*d*_6_): *δ*_C_ 14.23 (CH_3_), 17.66 (CH_2_), 18.49 (CH_2_), 31.87 (methine-C), 88.38, 104.85, 112.26, 113.41, 117.50, 117.80, 120.02, 120.60, 120.93, 122.27, 125.69, 126.36, 127.83, 128.15, 130.74, 132.40, 136.28, 148.24, 156.78, 163.09, 188. 11 (C=O); MS (EI, 70 ev) *m*/*z* (%): 398 [M]^+^, 355, 337, 382, 214, 184, 160, 144 (100), 131, 116, 103, 89, 77, 63.

## Author contributions

Amirhossein Khanmohammadi conducted the synthesis and characterization; Abolfazl Olyaei supervision, project administration, scientific advice, writing – review & editing; Mahdieh Sadeghpour writing – original draft and editing. All authors reviewed and approved the manuscript.

## Conflicts of interest

There are no conflicts to declare.

## Supplementary Material

RA-016-D6RA02182D-s001

## Data Availability

Experimental data and characterization details for all compounds are provided in the article. ^1^H-NMR and ^13^C-NMR spectra for compounds 4a–l are available in the supplementary information (SI). Supplementary information is available. See DOI: https://doi.org/10.1039/d6ra02182d.
