# Supplementary material for: Regioselective green synthesis of some novel indole-substituted 1H-benzo[f]chromenes via one-pot three-component reactions in water–ethanol media
Source: RSC Adv. 2026 Apr 2;16(20):17815–24. doi: 10.1039/d6ra02182d (PMC13044927; doi:10.1039/d6ra02182d)
Supplement: RA-016-D6RA02182D-s001 [file RA-016-D6RA02182D-s001.pdf]

Supplementary Information (SI) for RSC Advances.
This journal is © The Royal Society of Chemistry 2026

Supplementary data

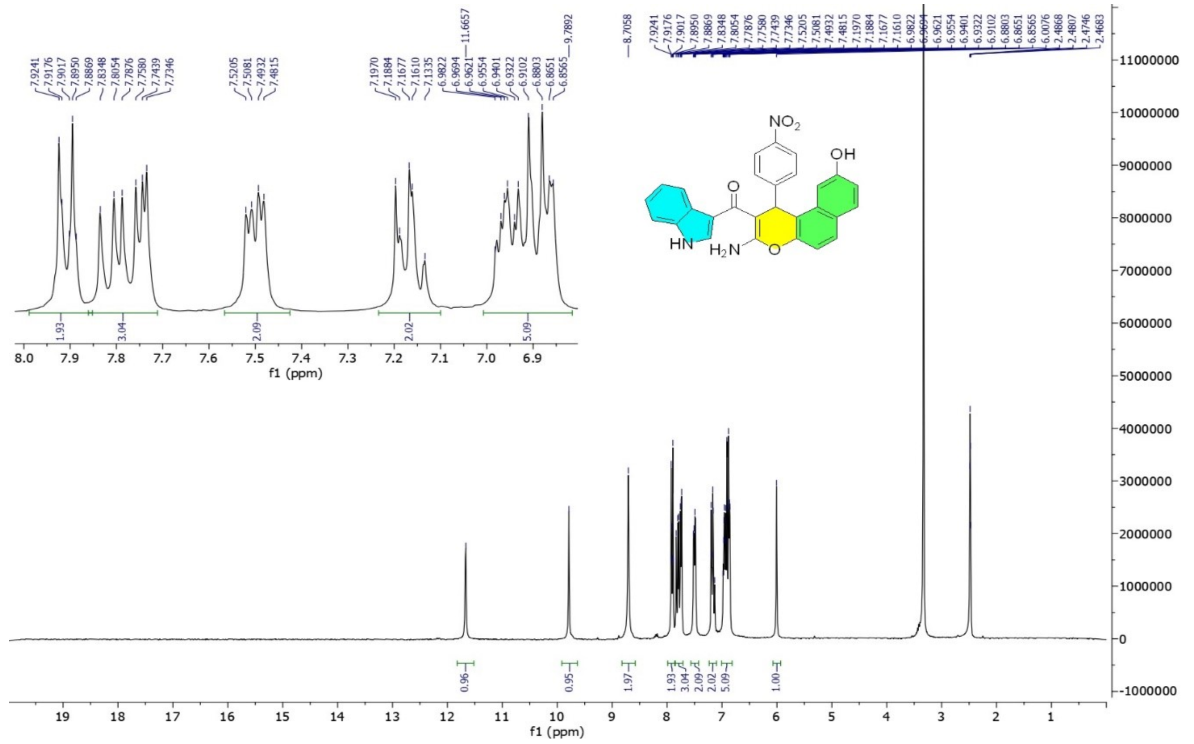

^{1}H-NMR spectrum of **4a** (300 MHz, DMSO-d_6)

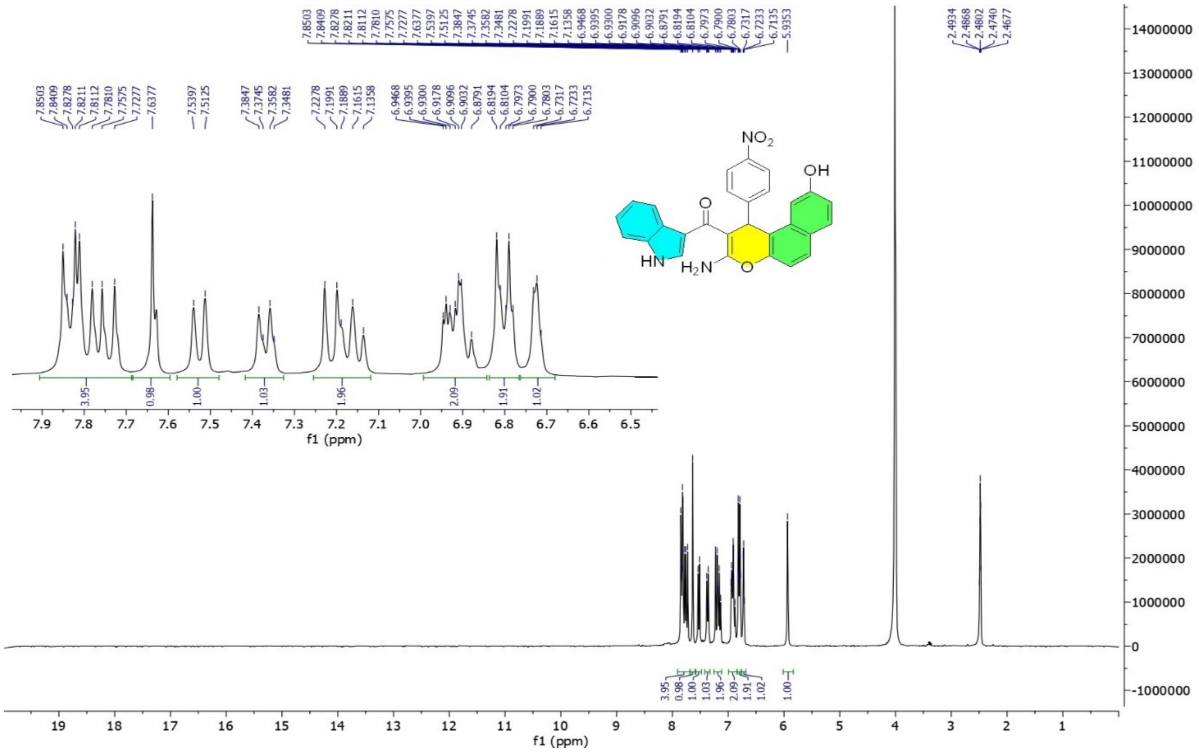

^{1}H-NMR spectrum of **4a** (300 MHz, DMSO-d_6 + D_2O)

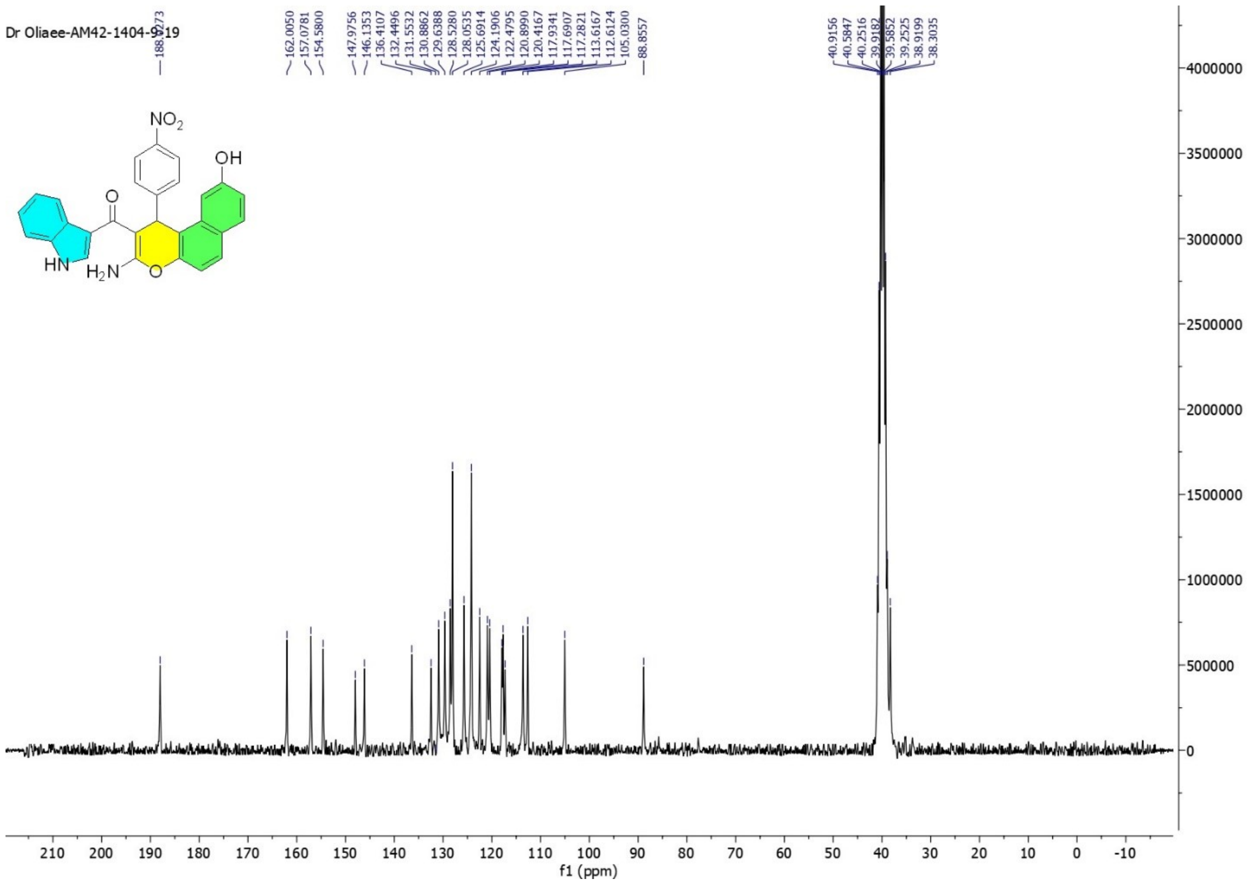

^{13}C-NMR spectrum of **4a** (75 MHz, DMSO-d_6)

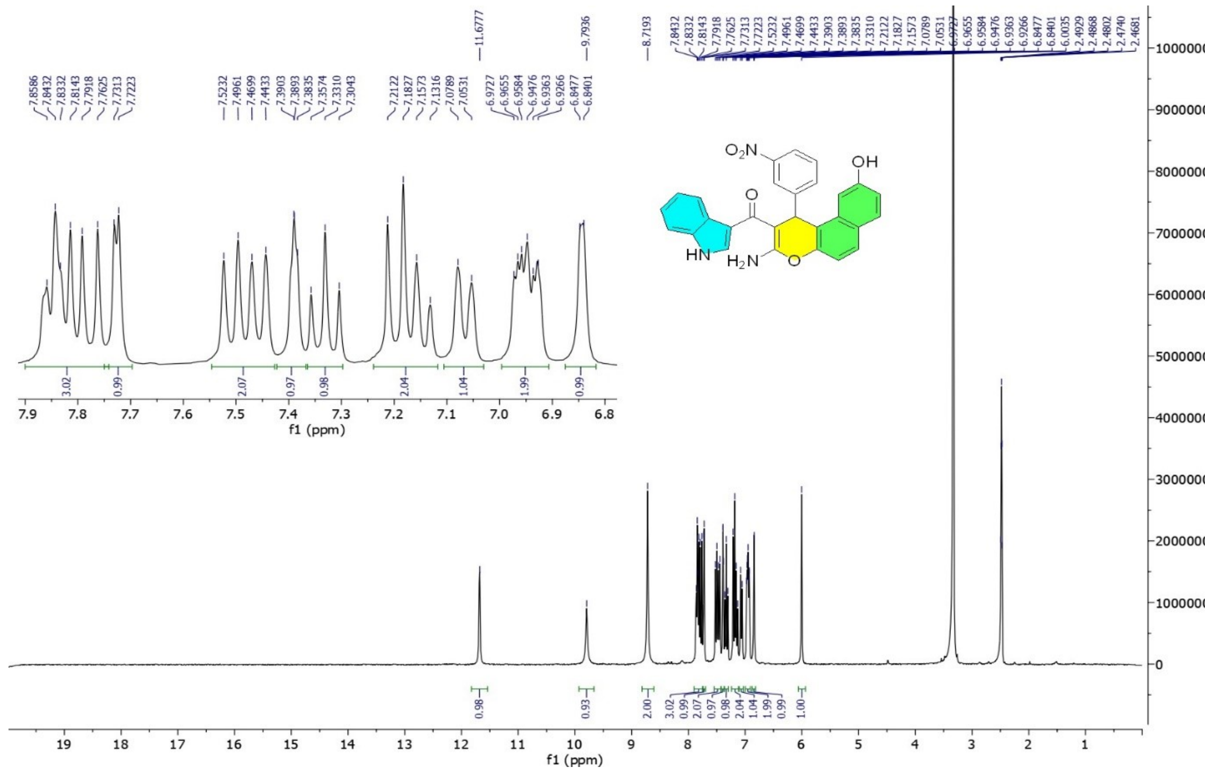

^{1}H-NMR spectrum of **4b** (300 MHz, DMSO-d_6)

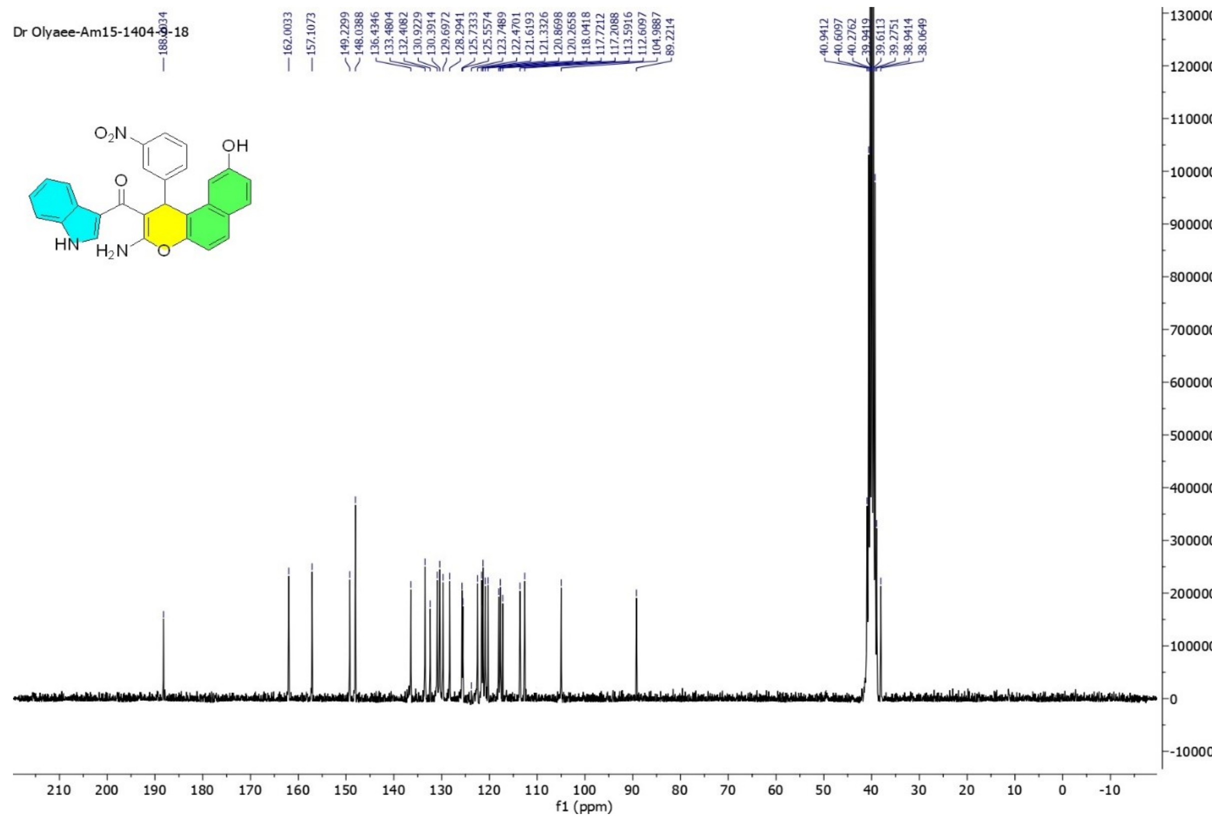

^{13}C-NMR spectrum of **4b** (75 MHz, DMSO-d_6)

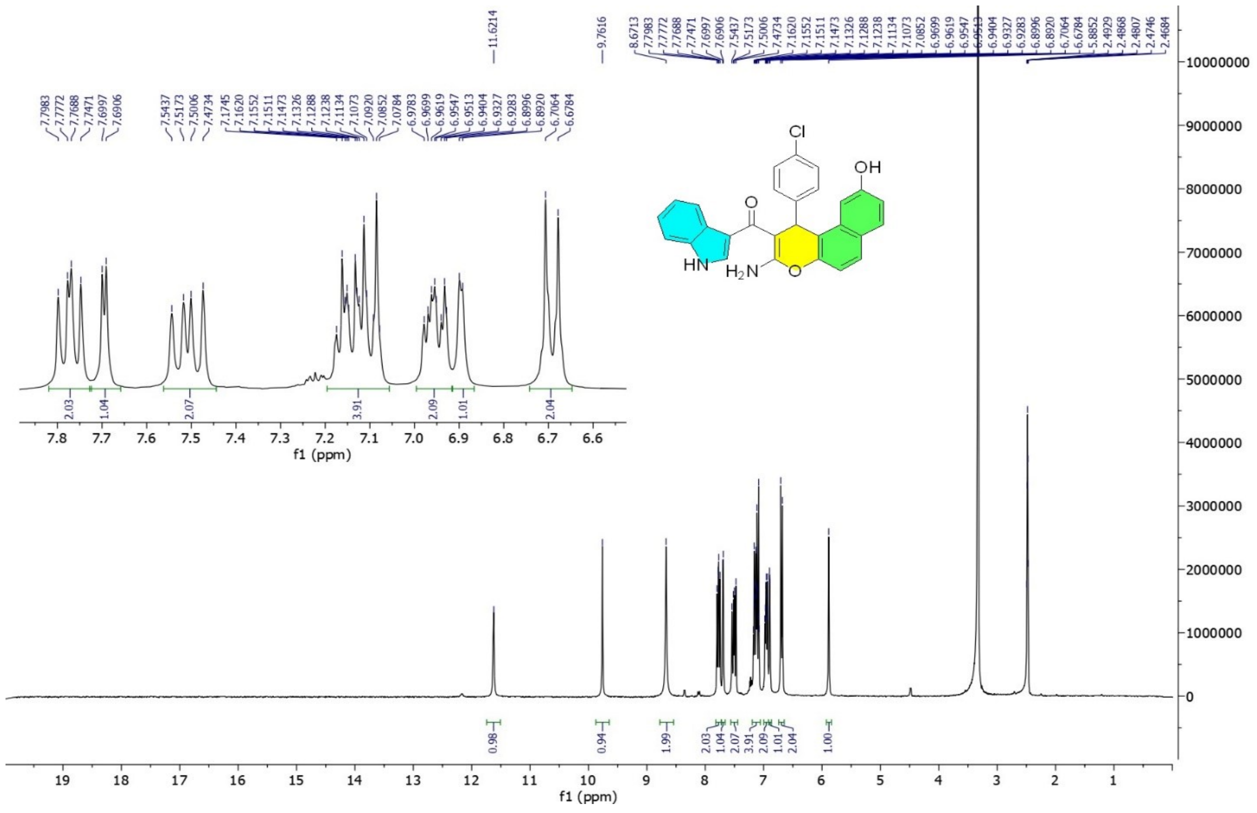

^{1}H-NMR spectrum of **4c** (300 MHz, DMSO-d_6)

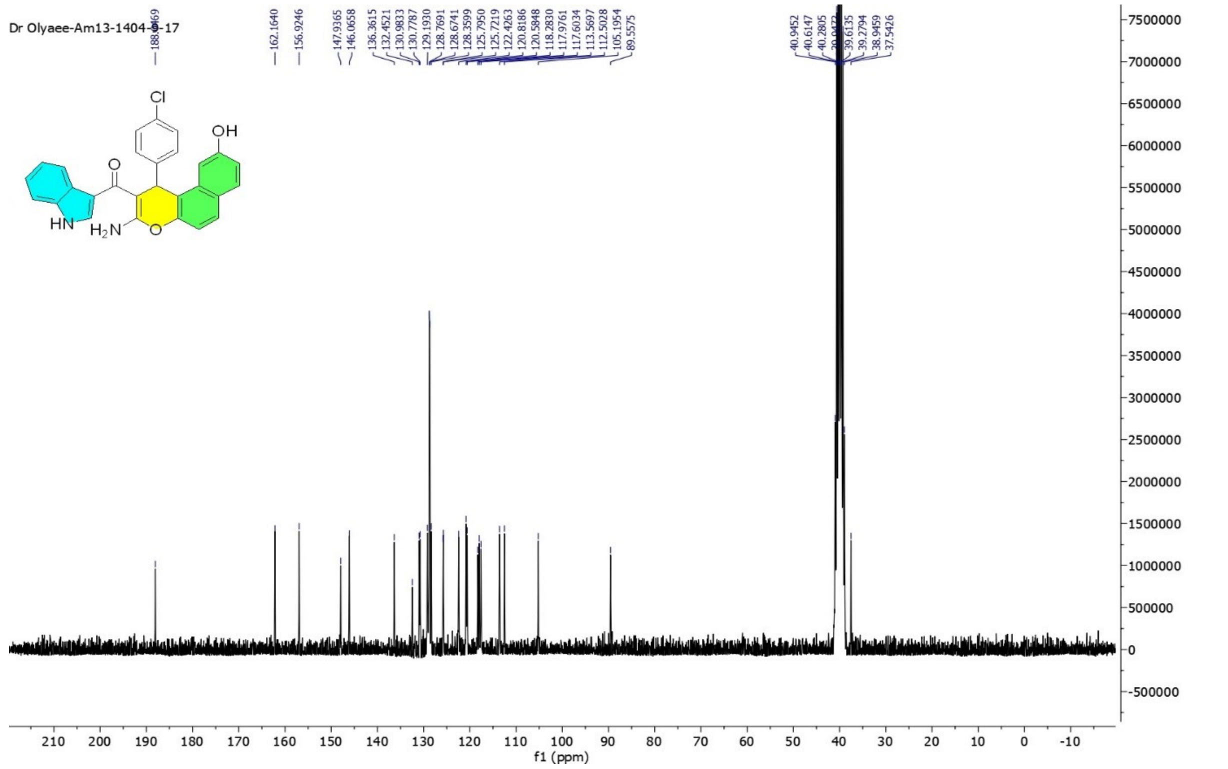

^{13}C-NMR spectrum of **4c** (75 MHz, DMSO-d_6)

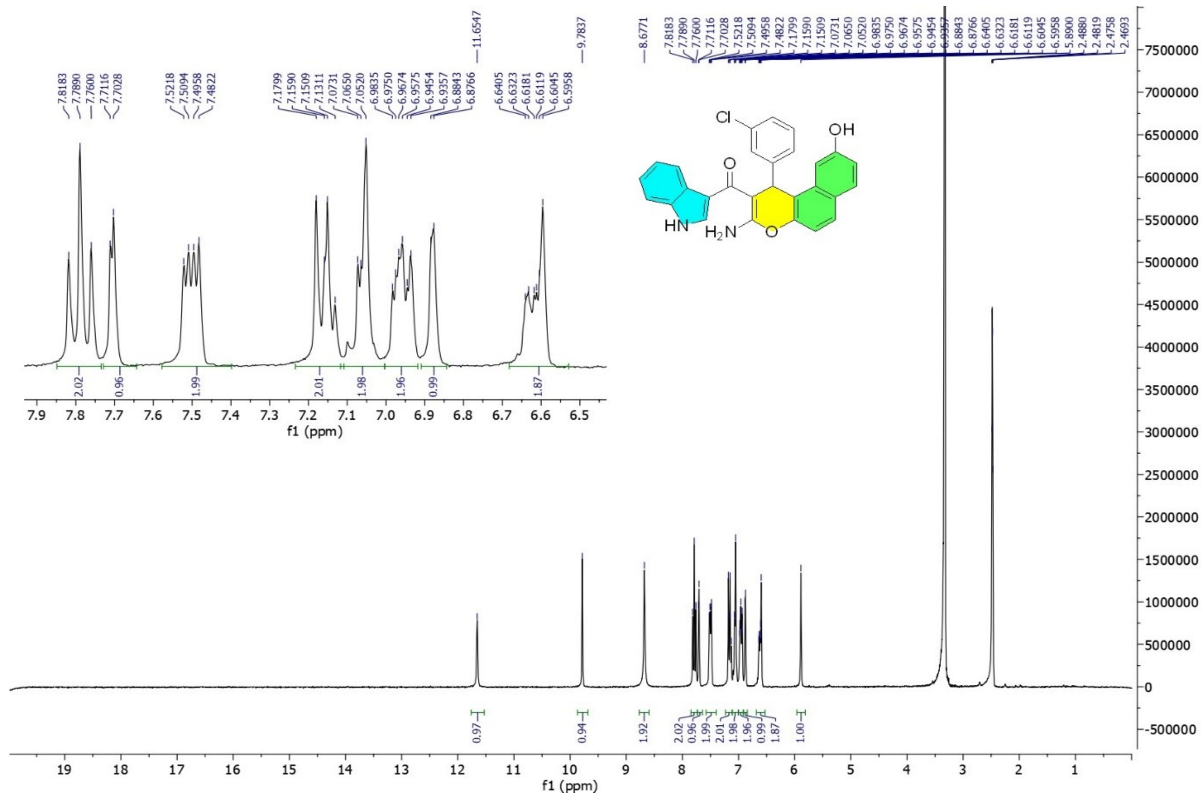

^{1}H-NMR spectrum of **4d** (300 MHz, DMSO-d_6)

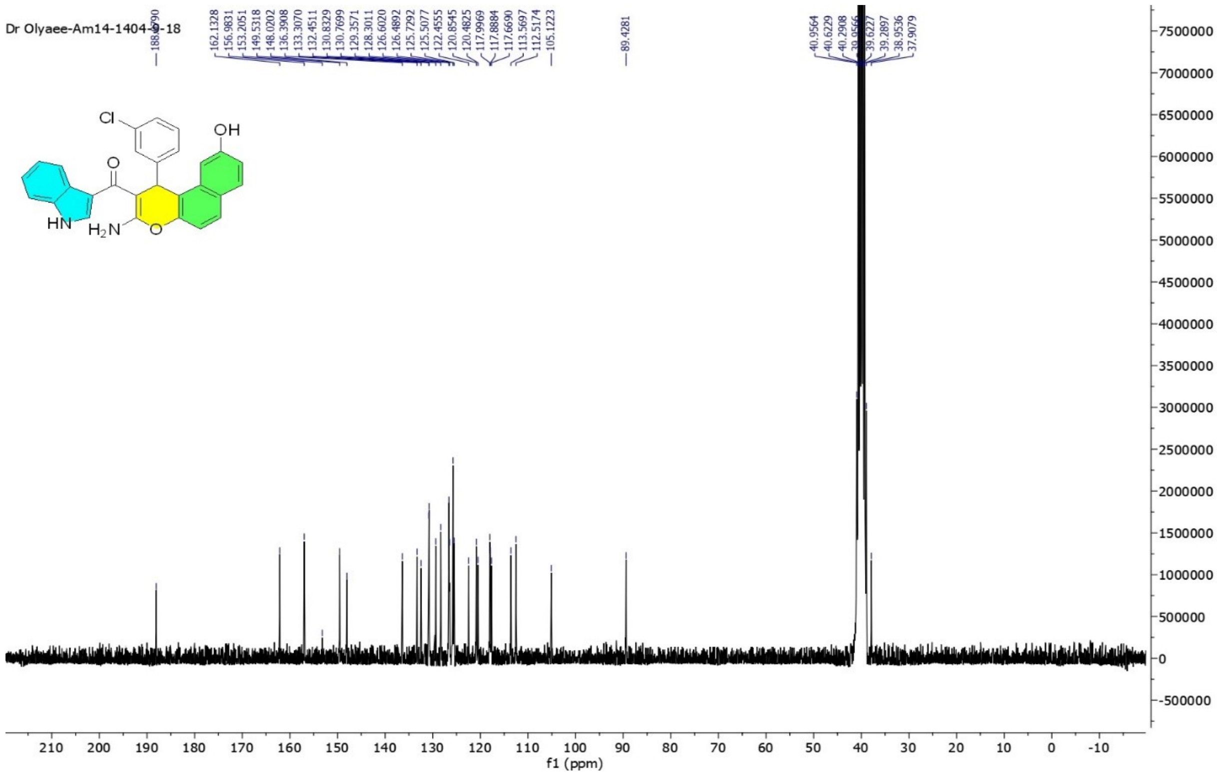

^{13}C-NMR spectrum of **4d** (75 MHz, DMSO-d_6)

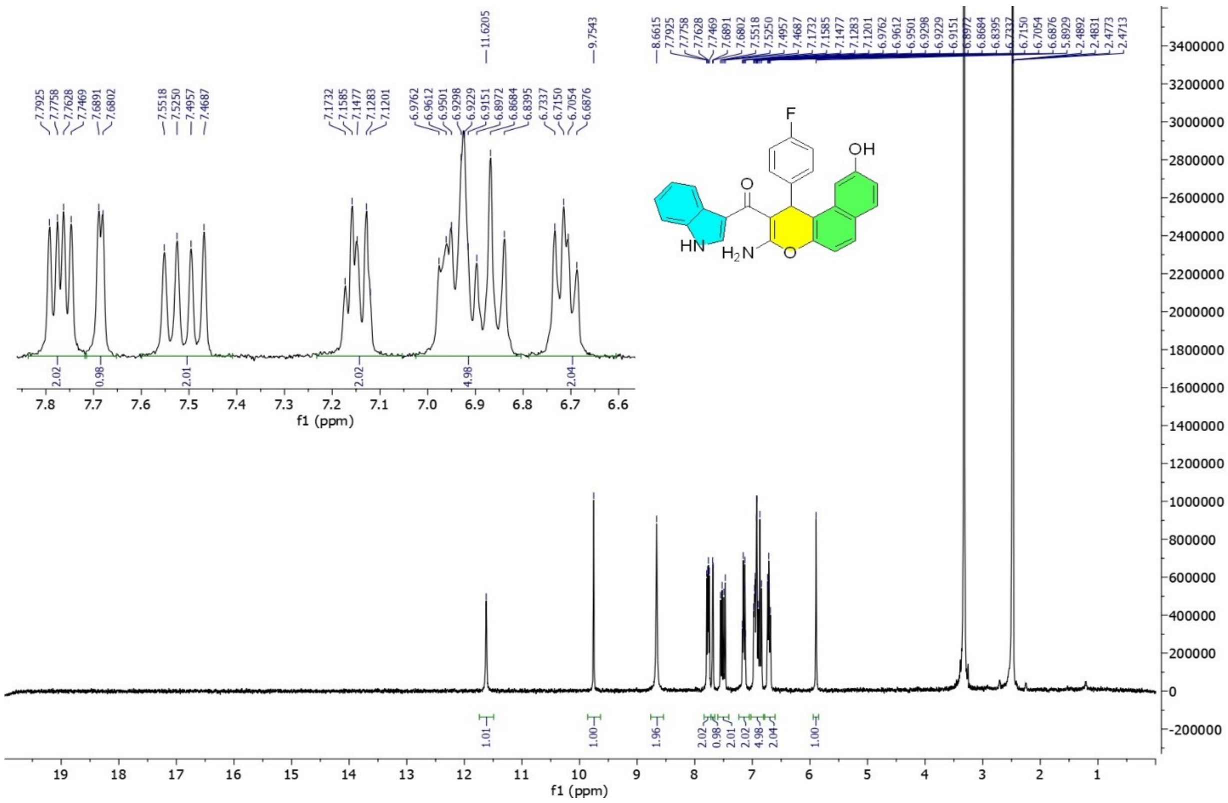

^{1}H-NMR spectrum of **4e** (300 MHz, DMSO-d_6)

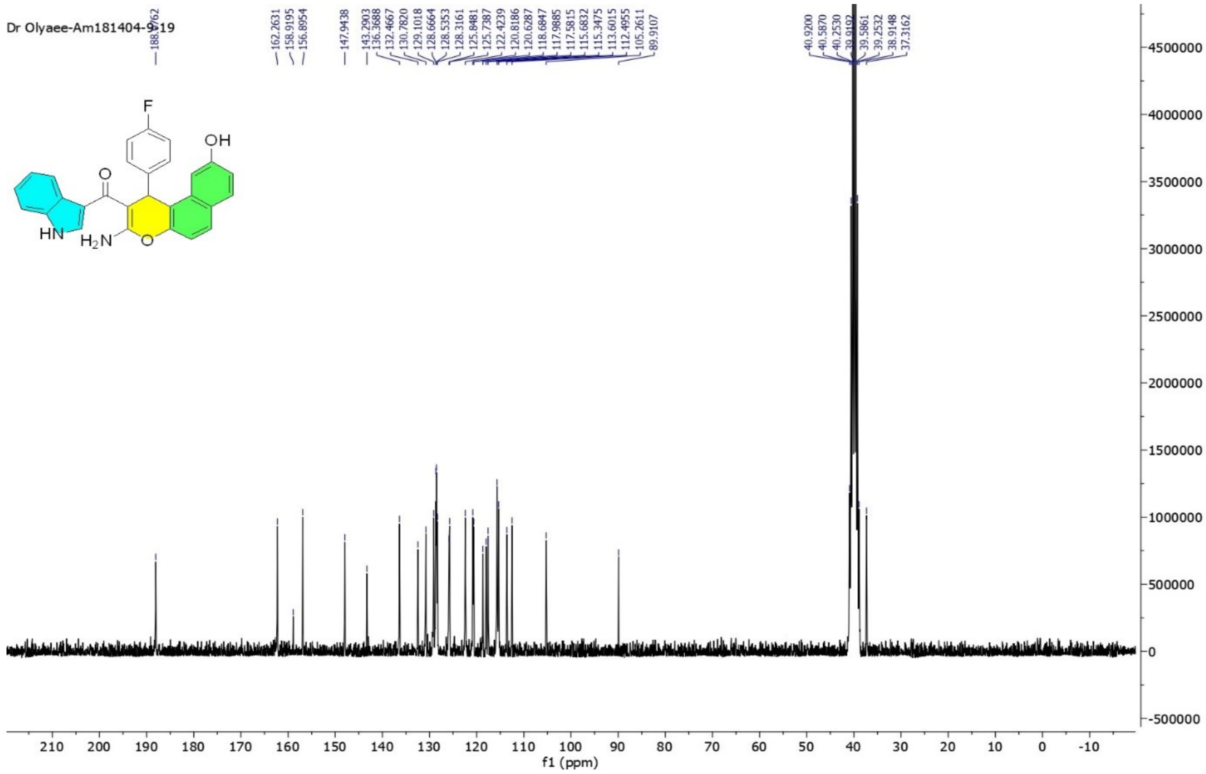

^{13}C-NMR spectrum of **4e** (75 MHz, DMSO-d_6)

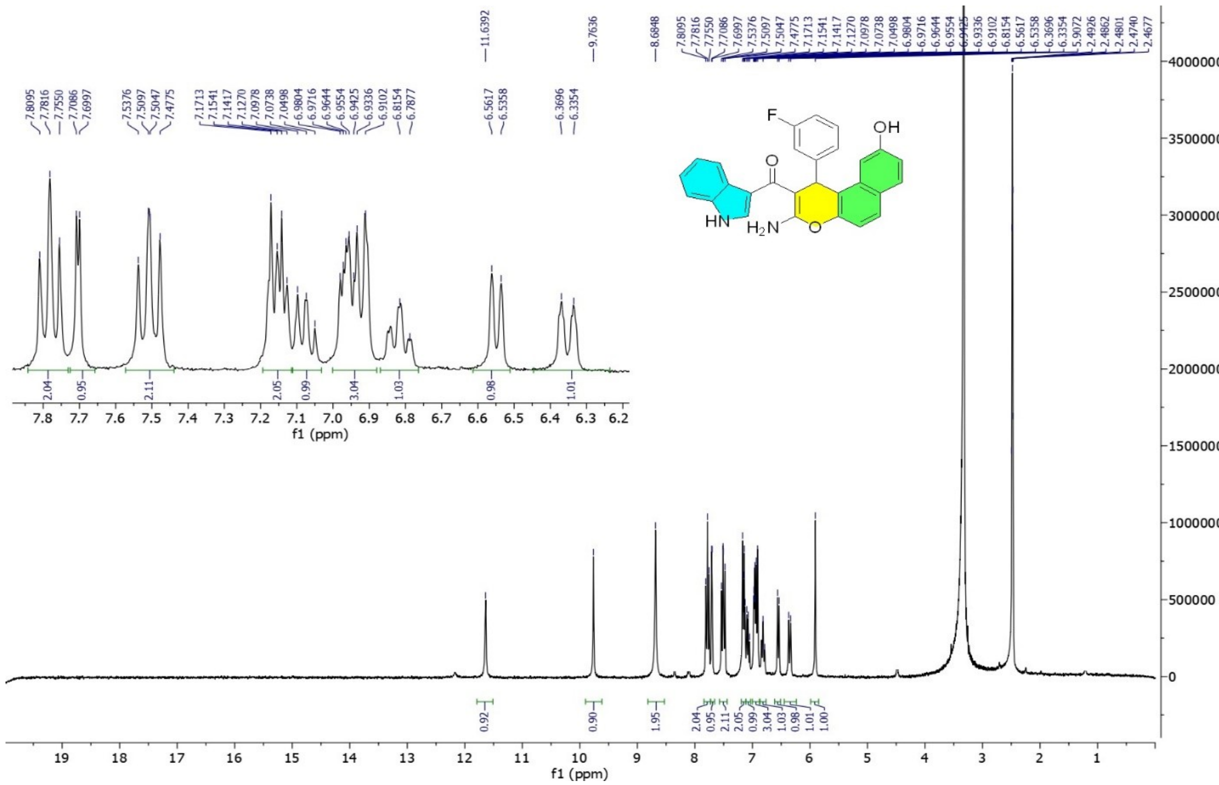

^{1}H-NMR spectrum of **4f** (300 MHz, DMSO-d_6)

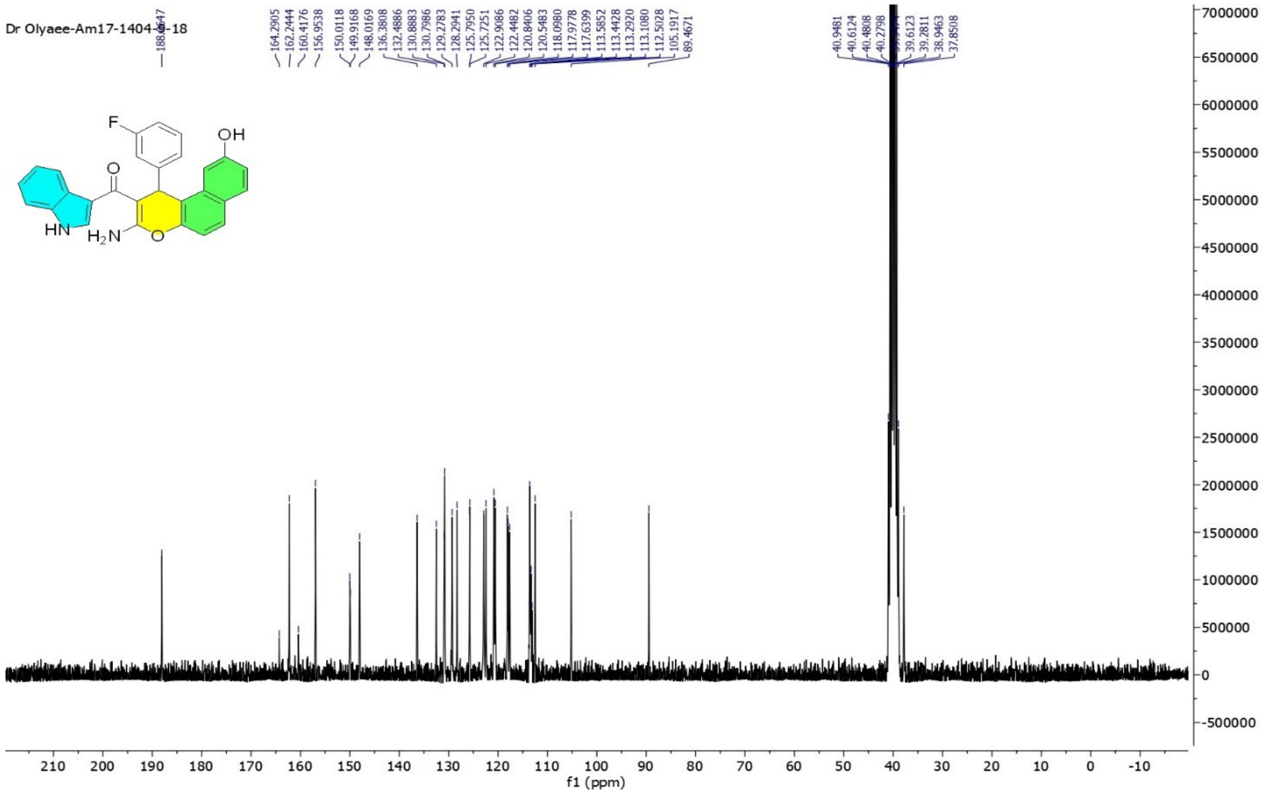

^{13}C-NMR spectrum of **4f** (75 MHz, DMSO-d_6)

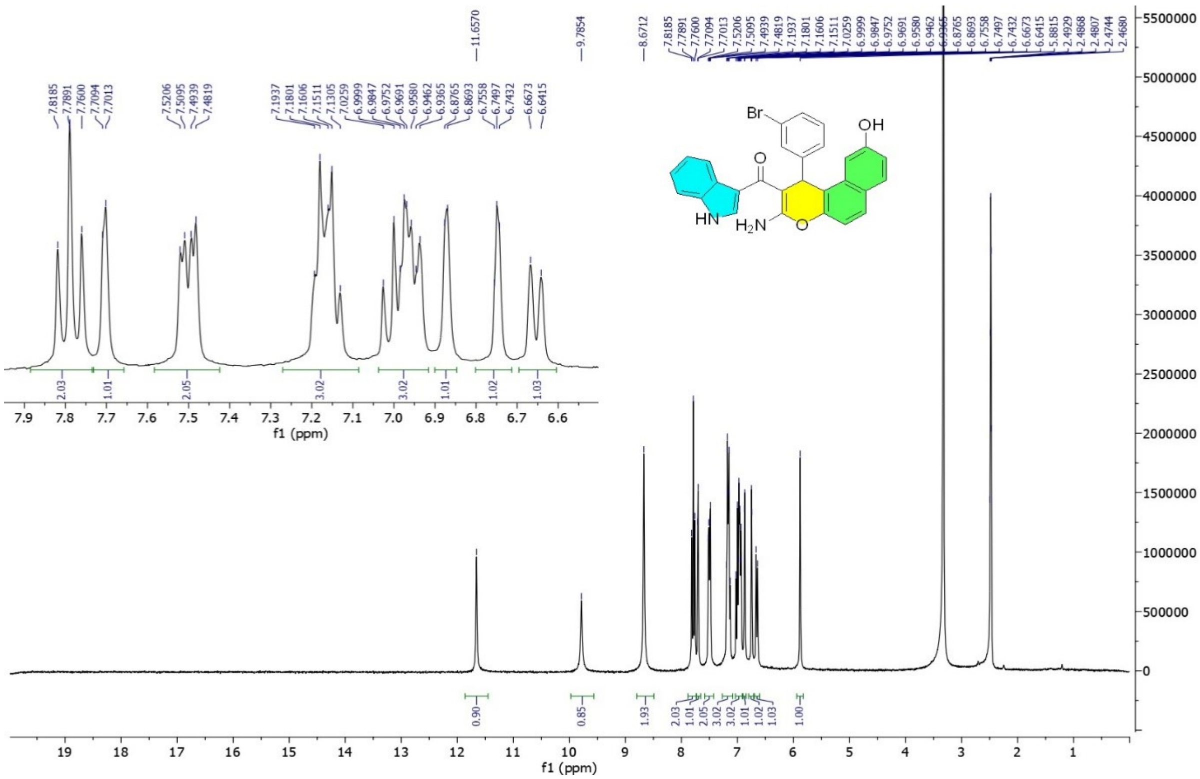

^{1}H-NMR spectrum of **4g** (300 MHz, DMSO-d_6)

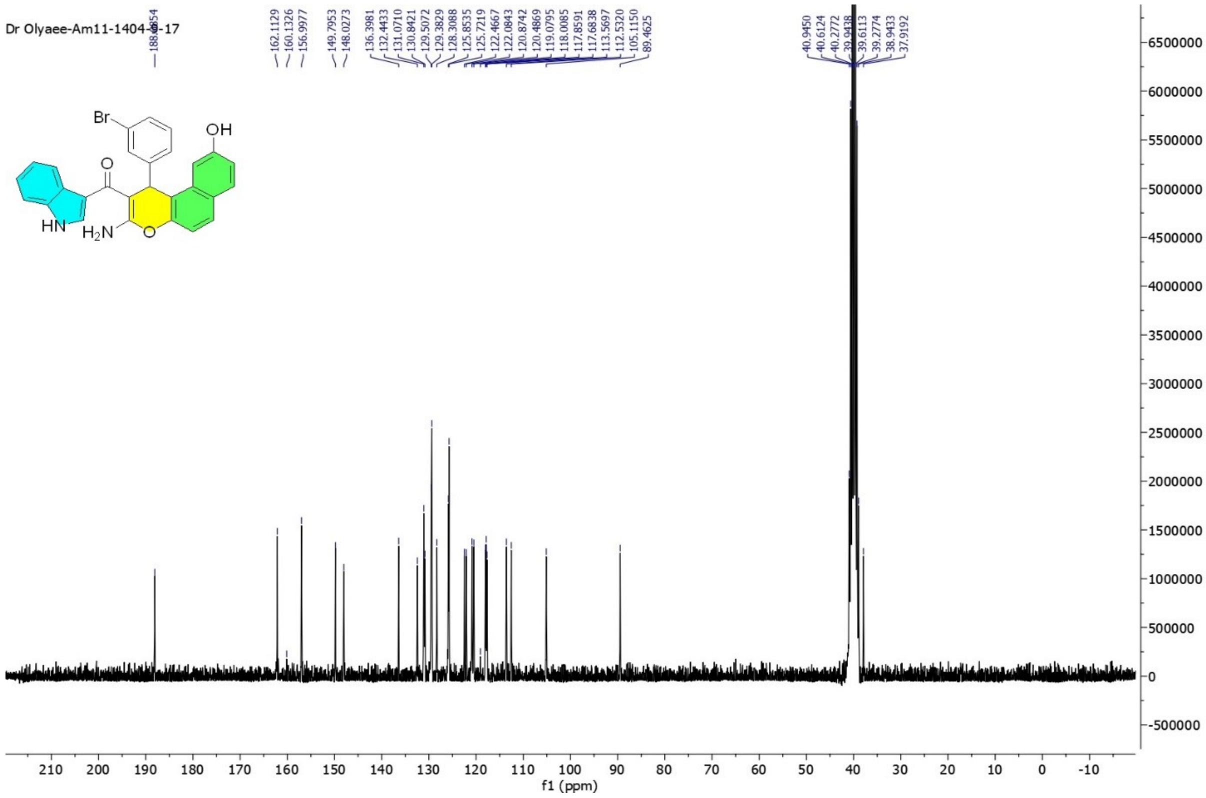

^{13}C-NMR spectrum of **4g** (75 MHz, DMSO-d_6)

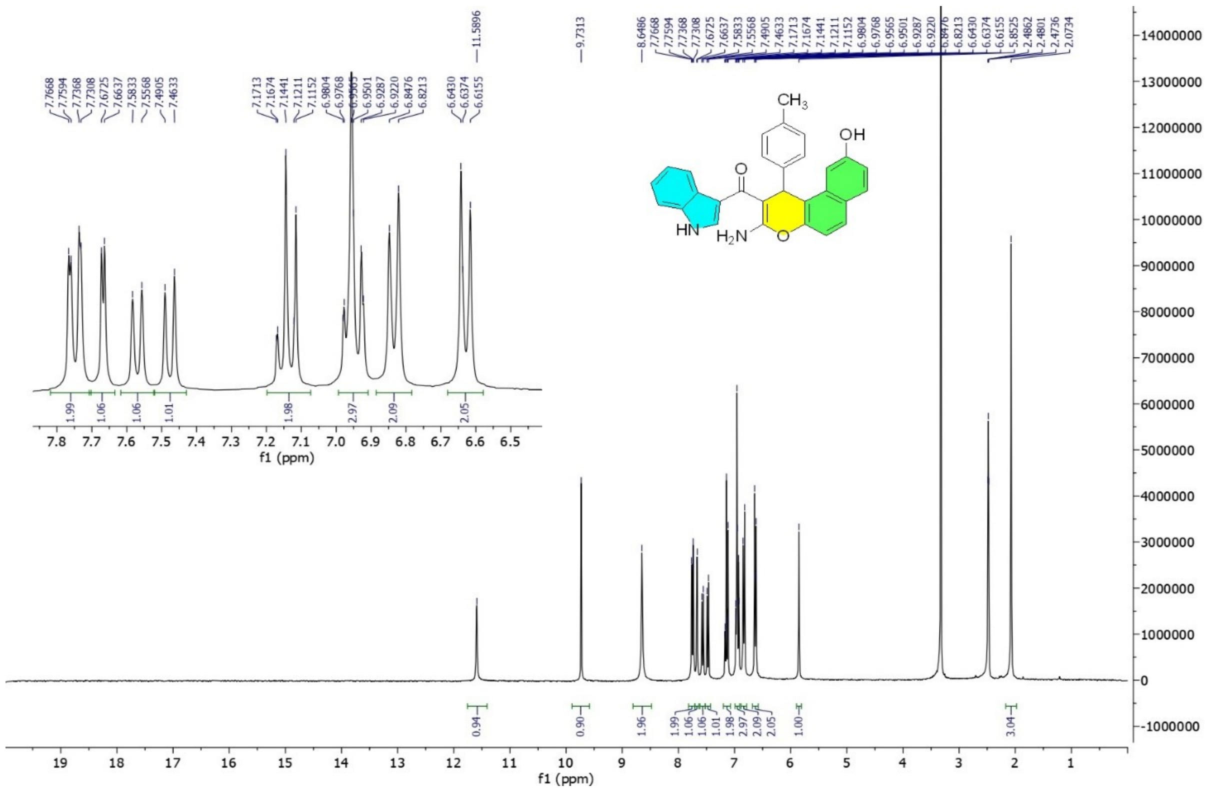

^{1}H-NMR spectrum of **4h** (300 MHz, DMSO-d_6)

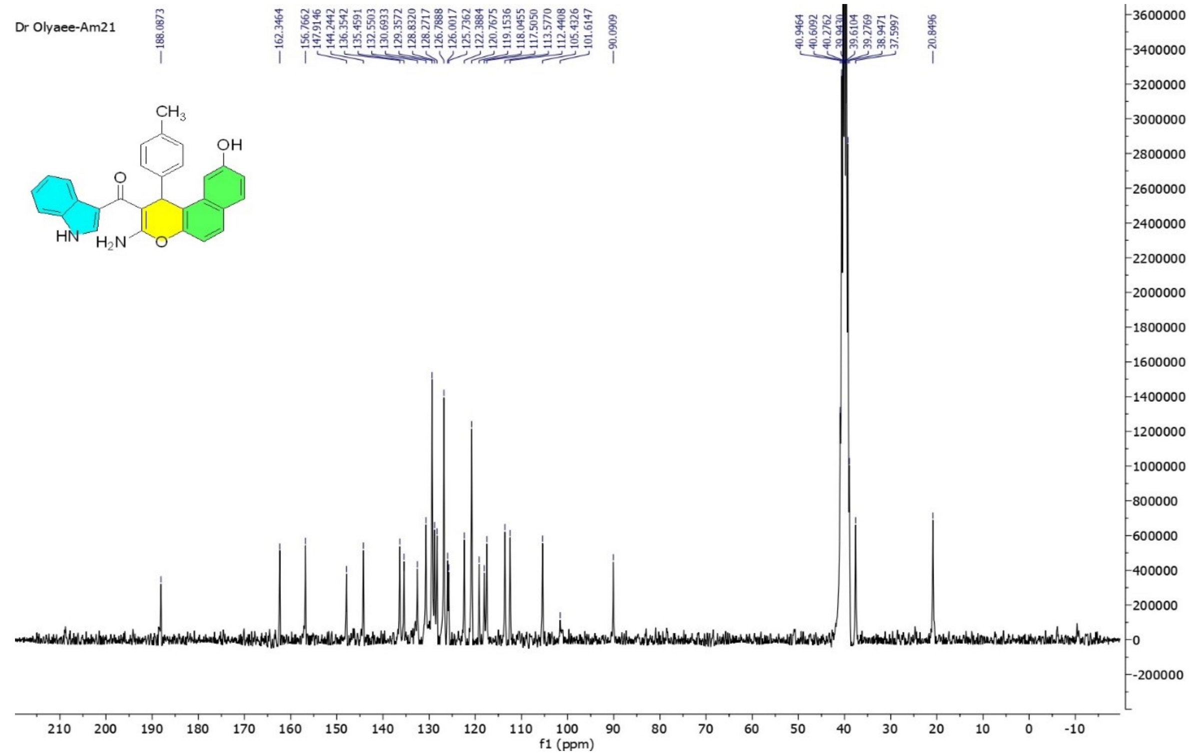

^{13}C-NMR spectrum of **4h** (75 MHz, DMSO-d_6)

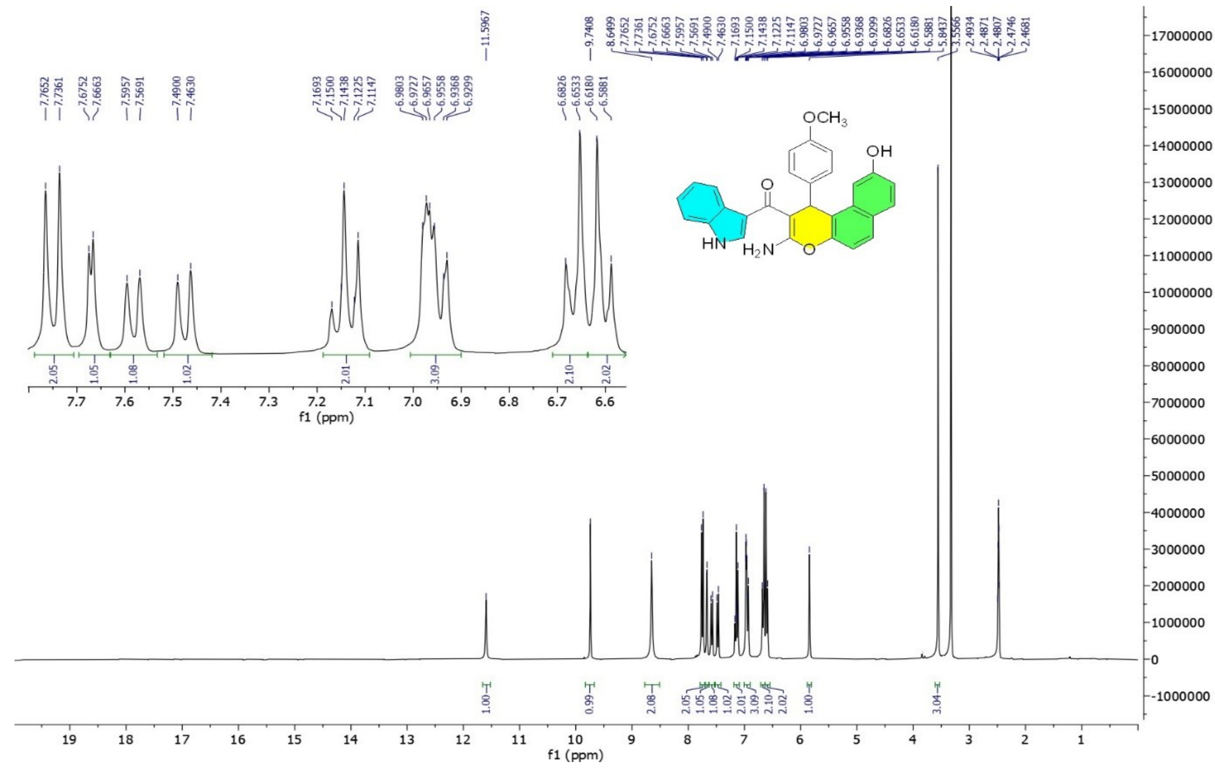

^{1}H-NMR spectrum of **4i** (300 MHz, DMSO-d_6)

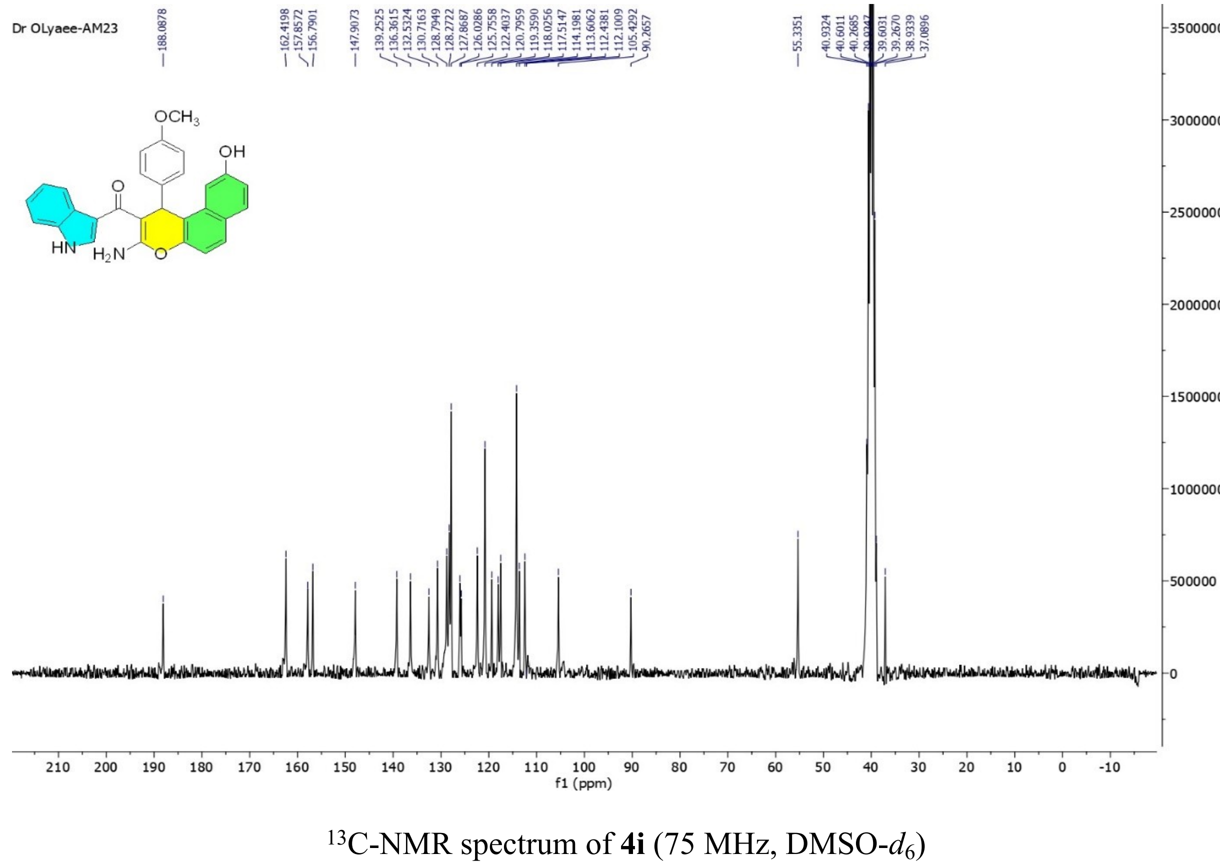

^{13}C-NMR spectrum of **4i** (75 MHz, DMSO-d_6)

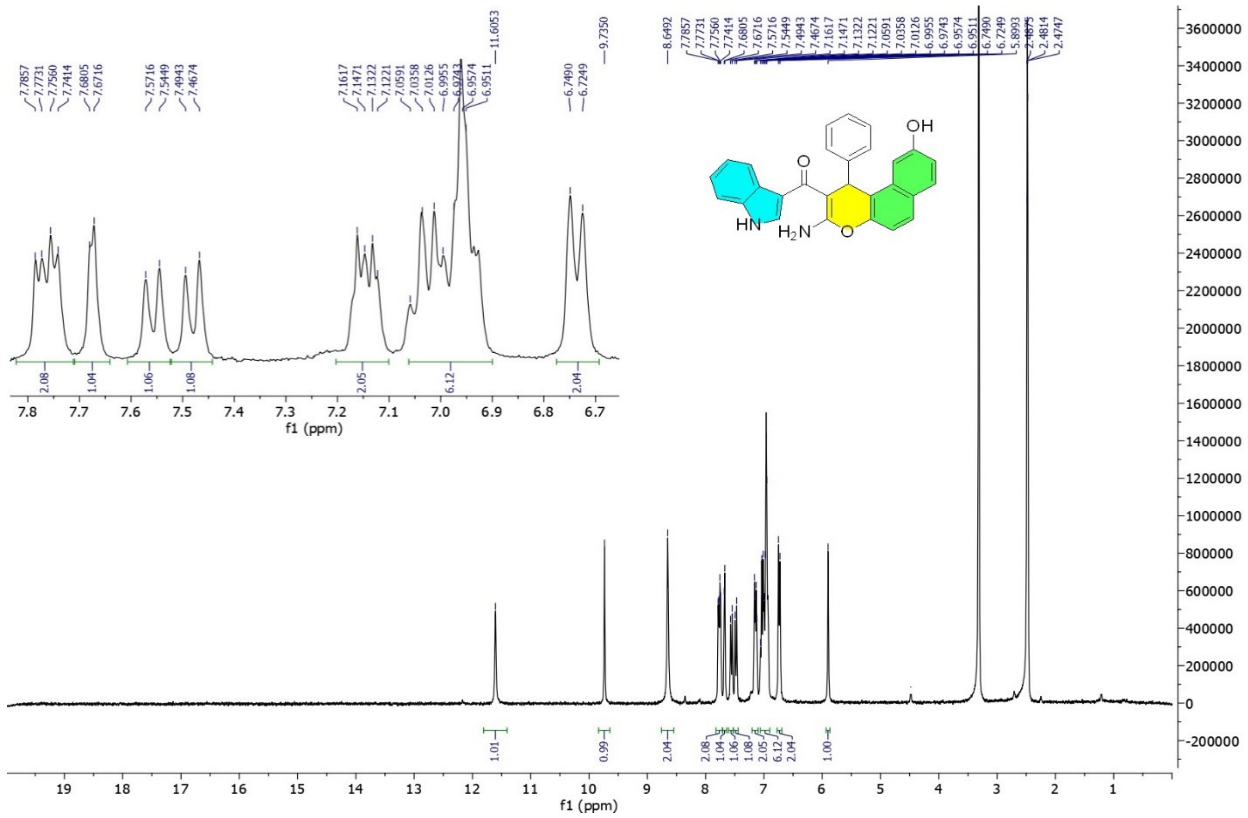

^{1}H-NMR spectrum of **4j** (300 MHz, DMSO-d_6)

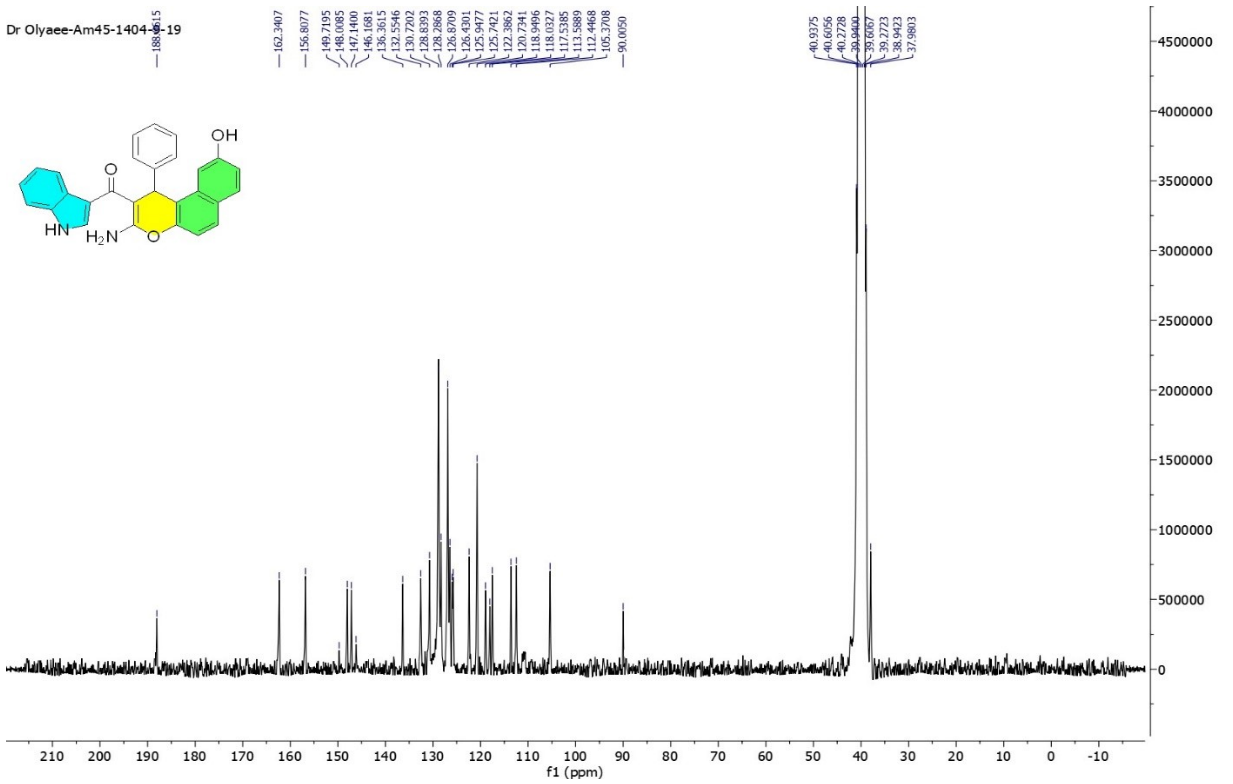

^{13}C-NMR spectrum of **4j** (75 MHz, DMSO-d_6)

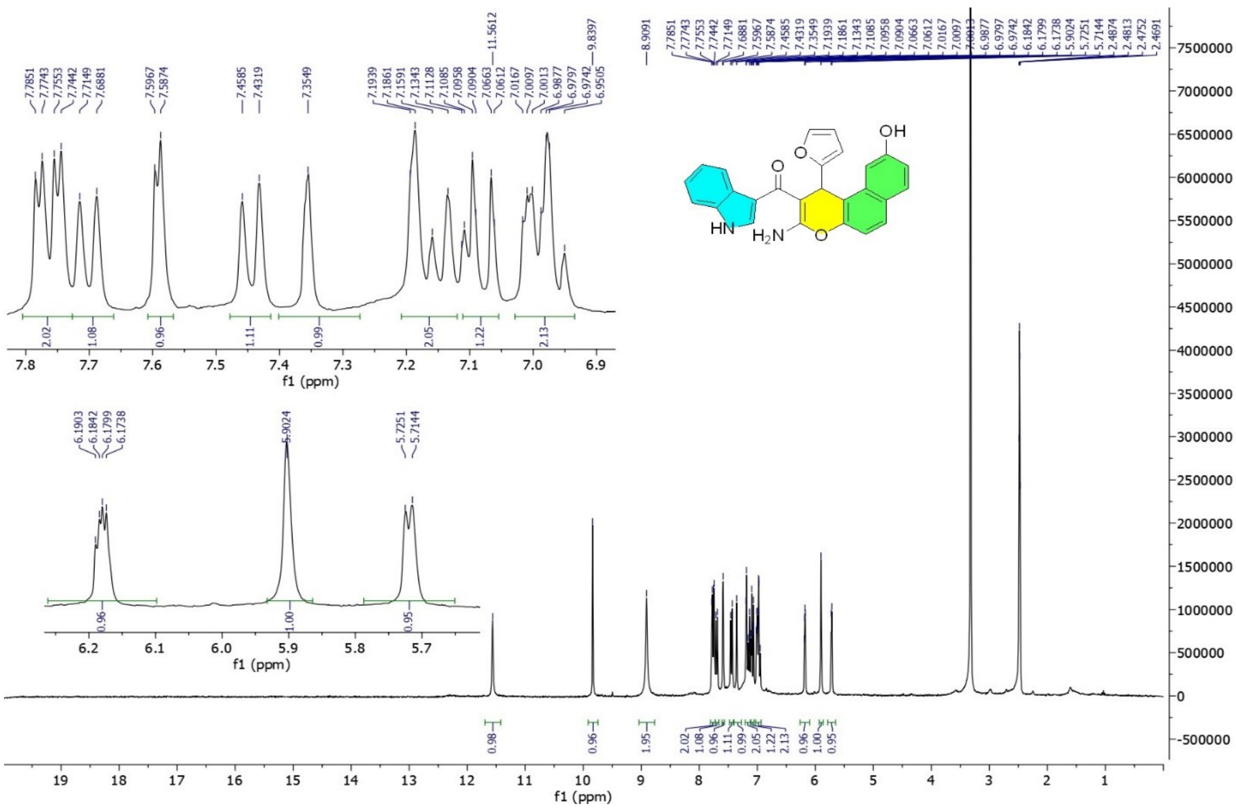

^{1}H-NMR spectrum of **4k** (300 MHz, DMSO-d_6)

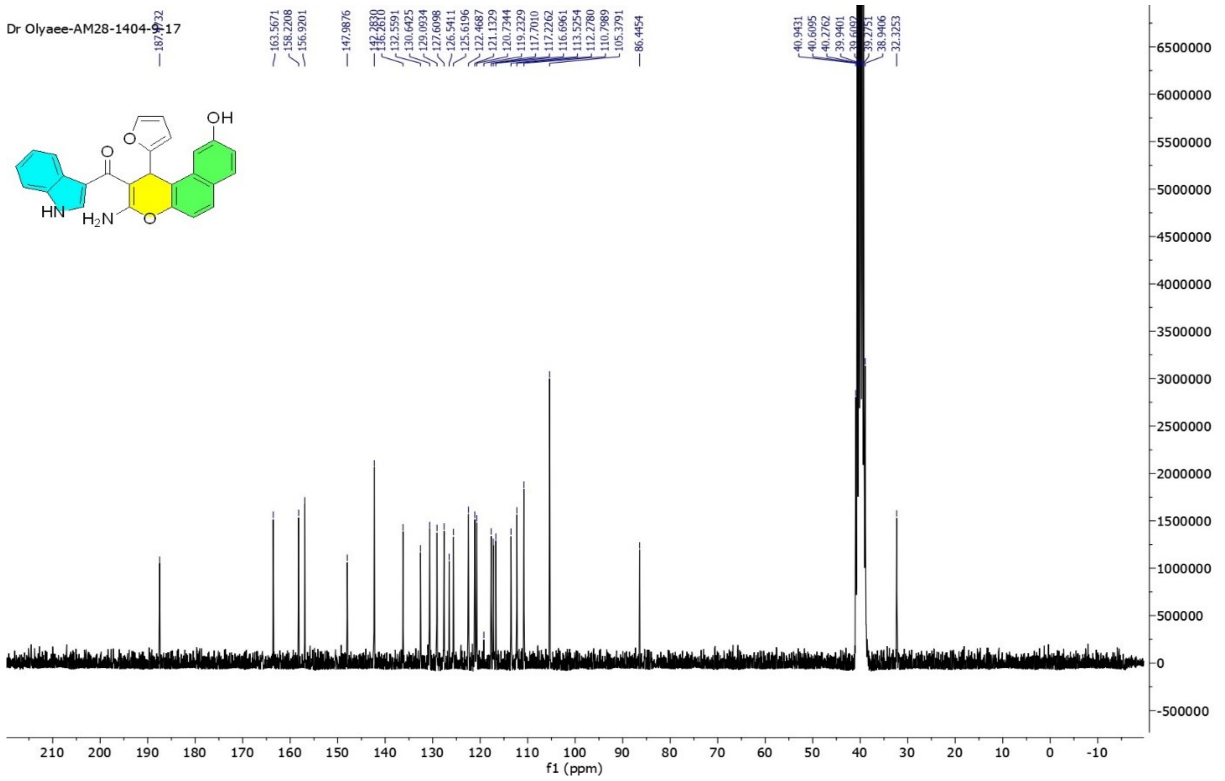

^{13}C-NMR spectrum of **4k** (75 MHz, DMSO-d_6)

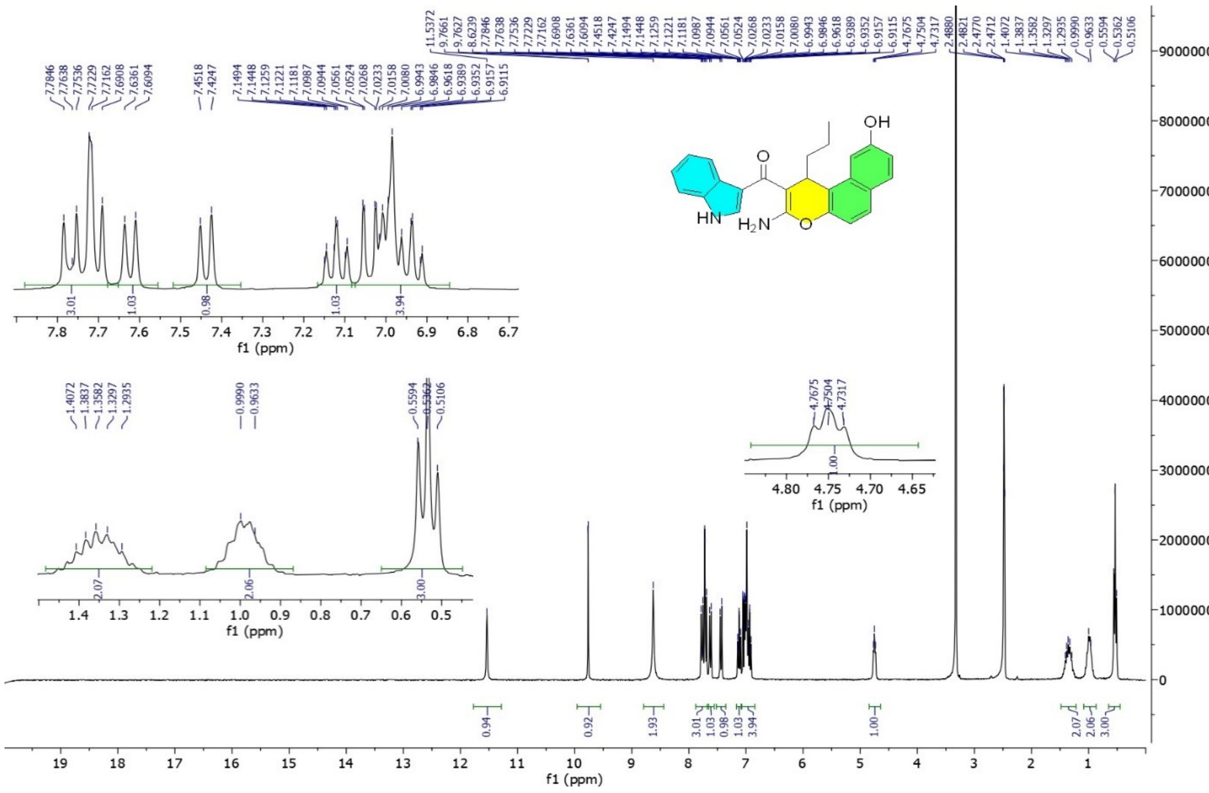

^{1}H-NMR spectrum of **4l** (300 MHz, DMSO-d_6)

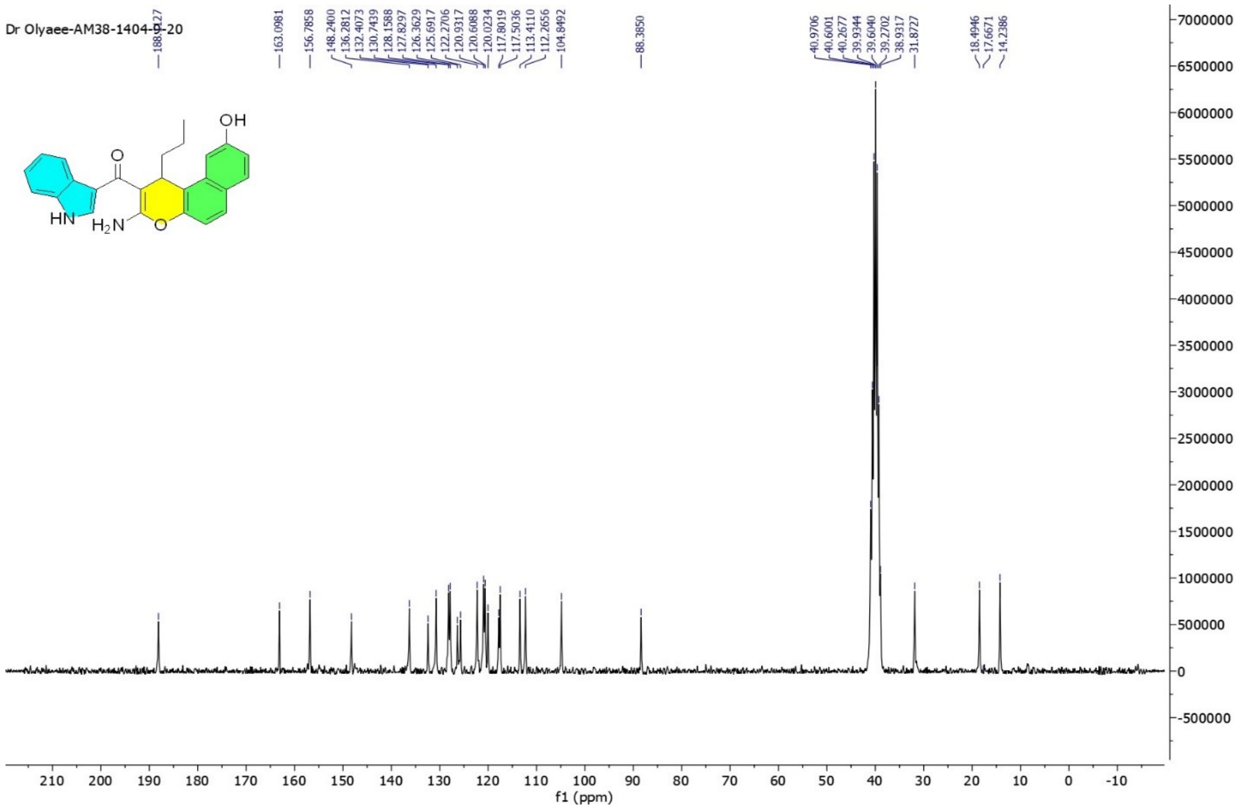

^{13}C-NMR spectrum of **4l** (75 MHz, DMSO-d_6)
